# Self-wetting triphase photocatalysis for effective and selective removal of hydrophilic volatile organic compounds in air

**DOI:** 10.1038/s41467-021-26541-z

**Published:** 2021-10-29

**Authors:** Fei He, Seunghyun Weon, Woojung Jeon, Myoung Won Chung, Wonyong Choi

**Affiliations:** 1 Division of Environmental Science and Engineering, Pohang University of Science and Technology (POSTECH), Pohang, 37673 Korea; 2 School of Health and Environmental Science, Korea University, Seoul, 02841 Korea

**Keywords:** Chemical engineering, Photocatalysis, Pollution remediation

## Abstract

Photocatalytic air purification is widely regarded as a promising technology, but it calls for more efficient photocatalytic materials and systems. Here we report a strategy to introduce an in-situ water (self-wetting) layer on WO_3_ by coating hygroscopic periodic acid (PA) to dramatically enhance the photocatalytic removal of hydrophilic volatile organic compounds (VOCs) in air. In ambient air, water vapor is condensed on WO_3_ to make a unique tri-phasic (air/water/WO_3_) system. The in-situ formed water layer selectively concentrates hydrophilic VOCs. PA plays the multiple roles as a water-layer inducer, a surface-complexing ligand enhancing visible light absorption, and a strong electron acceptor. Under visible light, the photogenerated electrons are rapidly scavenged by periodate to produce more •OH. PA/WO_3_ exhibits excellent photocatalytic activity for acetaldehyde degradation with an apparent quantum efficiency of 64.3% at 460 nm, which is the highest value ever reported. Other hydrophilic VOCs like formaldehyde that are readily dissolved into the in-situ water layer on WO_3_ are also rapidly degraded, whereas hydrophobic VOCs remain intact during photocatalysis due to the “water barrier effect”. PA/WO_3_ successfully demonstrated an excellent capacity for degrading hydrophilic VOCs selectively in wide-range concentrations (0.5−700 ppmv).

## Abstract (web summary)

Photocatalytic air purification is promising but it calls for more efficient photocatalytic materials and systems. Here, the authors report a strategy to introduce an in-situ water layer on WO_3_ by coating hygroscopic periodic acid that effectively remove hydrophilic volatile organic compounds.

## Introduction

Volatile organic compounds (VOCs) are major components of air pollution, which significantly deteriorate air quality and seriously affect human health^1–3^. A common control method for VOCs is adsorption using porous medium (e.g., activated carbon, zeolite, MOFs, etc.)^4,5^, but their equilibrium adsorption capacity decreases significantly with lowering VOCs concentration^6^. Photocatalysis is widely considered as a promising method for air purification because of its ability to operate under ambient temperature and pressure conditions and to degrade and mineralize VOCs^7–9^. Photocatalytic degradation (PCD) maintains its removal efficiency even in the low concentration range^3,10^, which is more advantageous to deal with VOCs in sub-ppm levels (e.g., indoor air)^11^. Considering that visible light takes a much higher proportion (~43%) of solar light than UV light (~4%) and a dominant portion of indoor light, it is essential to develop visible-light-responsive photocatalysts for practical application of air purification^12^. However, the performance of visible-light-driven photocatalysts is generally much lower than UV photocatalysis and needs significant improvements to satisfy the requirements for practical air purification^13^. As the PCD of VOCs is initiated mainly by hydroxyl radical (•OH) attack^14–16^, an efficient way of enhancing visible light PCD is to facilitate the generation of •OH.

WO_3_ is one of the most frequently investigated photocatalysts with notable visible light activity (*E*_g_ ≈ 2.8 eV)^10,17,18^, which is also stable in oxidative and acidic condition^19^. Although its valence band (VB) edge potential (at about 3.0 V_NHE_) is positive enough to generate OH radicals (•OH/H_2_O, *E*^0^ = +2.8 V_NHE_)^13^, its conduction band (CB) edge potential (~0.4 V_NHE_)^10^ is not negative enough to make CB electrons scavenged by O_2_ via a single-electron transfer (e.g., O_2_/O_2_•^−^, *E*^0^ = −0.33 V_NHE_; O_2_/HO_2_•, *E*^0^ = −0.05 V_NHE_)^13,20^. As a result, the photocatalytic activity of WO_3_ is highly limited due to the rapid charge recombination^21,22^. Many attempts have been made to increase the visible light activity of WO_3_ and one of the most effective methods is to load Pt nanoparticles as a co-catalyst^23^, which enables the multi-electron reduction of O_2_ to H_2_O_2_ or H_2_O at the WO_3_ CB edge potential (O_2_/H_2_O_2_, *E*^0^ = +0.68 V_NHE_; O_2_/H_2_O, *E*^0^ = +1.23 V_NHE_)^24–26^ with facilitating the charge separation and subsequently OH radical production^27^. However, the use of expensive Pt cocatalyst limits its practical applications^28^, which calls for a more economical method utilizing cheaper material. In typical PCD mechanisms working on the gas–solid interface, photogenerated holes most likely react with adsorbed water molecules (or surface hydroxyl groups) to form •OH^11,29^. Water molecules are not only a source of •OH but also an adsorbent competing with VOCs for the surface sites^30,31^; the dual effects of water vapor affect the PCD of VOCs differently depending on the hydrophilic and hydrophobic nature of VOCs^32^.

In this work, we propose to utilize water in a uniquely different way for the removal of VOCs by introducing an in situ water layer on the photocatalyst surface in ambient air. The presence of a thin surface water layer selectively solubilizes and concentrates hydrophilic VOCs in it, which subsequently facilitates their PCD. To achieve this, periodic acid (PA, HIO_4_·2H_2_O) as a highly hygroscopic substance was employed to induce an in situ water layer formed between the photocatalyst surface and air phase; as a result, the gas–solid interface can be transformed into a gas–liquid–solid (triphase) interface. In addition, the highly oxidizing PA^33,34^ has the potential to serve as a strong scavenger of CB electrons, which should enhance the formation of •OH via the hole transfer. Herein, the PA/WO_3_ system was employed for the PCD of several VOCs to propose a concept of PA-assisted photocatalysis that employs the in situ formation of the water layer on the photocatalyst surface. The low-cost PA-loaded WO_3_ exhibits even higher activity than Pt-loaded WO_3_ for the PCDs of hydrophilic VOCs. This presents a cost-effective technology for high-performance selective degradation of hydrophilic VOCs.

## Results

### Enhanced activities of PA-coated photocatalysts

**Fig. 1:**
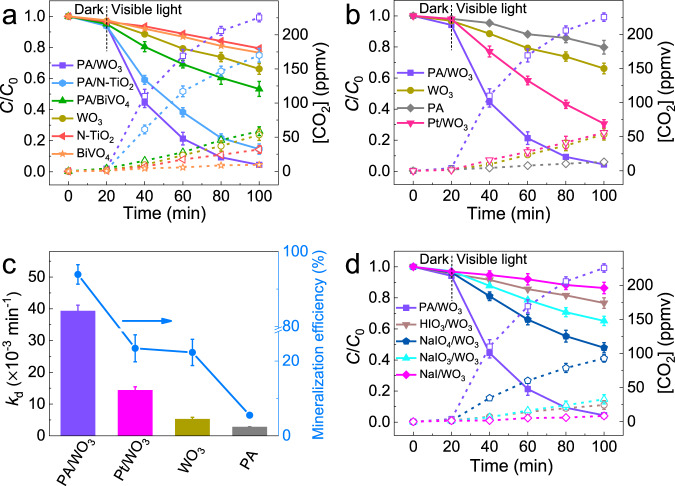
Visible light-driven PCD of acetaldehyde (AA). **a** The time profiles of the PCD of AA and the accompanying production of CO_2_ on bare and PA-treated WO_3_, N-TiO_2_, and BiVO_4_. **b** The PCD comparison between PA/WO_3_ and Pt/WO_3_. **c** The PCD rate constant (*k*_d_) and the mineralization efficiency after 80 min reaction for PA/WO_3_, Pt/WO_3_, bare PA, and WO_3_. **d** PCD activities of WO_3_ treated with different iodine reagents. The dashed lines with open symbols represent the CO_2_ concentration generated from AA degradation. Error bars are defined as standard deviation. Experimental conditions: [AA]_0_ = 120 ppmv; visible light (*λ* > 420 nm) intensity of 2.2 mW/cm^2^; sample amount of 50 mg; RH 65%; reaction temperature of 30 °C.

PA was combined with three common visible-light photocatalysts (BiVO_4_, N-TiO_2_, and WO_3_), and their photocatalytic activities were tested for acetaldehyde (AA) degradation in a closed-circulation reactor (Supplementary Fig. 1) under visible light (*λ* > 420 nm). Each PCD test consisted of a dark circulation period (20 min) for adsorption equilibrium and the following irradiation period. Figure 1a shows the degradation of AA and the accompanying production of CO_2_ over bare and PA-treated WO_3_, N-TiO_2_, and BiVO_4_. The PCD rate constant (*k*_d_), removal efficiency, and mineralization efficiency are summarized in Supplementary Table 1. The photocatalytic activities of WO_3_, N-TiO_2_, and BiVO_4_ were significantly improved by the PA treatment among which PA/WO_3_ exhibited the highest PCD activity. PA also has a similar PCD-promoting effect for TiO_2_ (P25) under UV irradiation (Supplementary Fig. 2), which confirms that the PA effect is the same regardless of the kind of photocatalysts. Interestingly, the photocatalytic activities of PA/P25 and PA/WO_3_ are similar under LED irradiation of 365 nm while PA/WO_3_ is far more active than PA/P25 under 460 nm LED irradiation (see Supplementary Figs. 2 and 3). It is particularly notable that PA/WO_3_ exhibited higher activity than Pt/WO_3_ which is one of the most active visible-light photocatalysts^10,23,27^. The optimal composition of PA/WO_3_ was tested by varying the mixing ratio of PA:WO_3_ which exhibits good activity for a wide range of PA:WO_3_ mass ratios between 2/3 and 3/2 (see Supplementary Fig. 4). The highest PCD activity was observed at the 1:1 mass ratio of PA:WO_3_, which was used in the preparation of PA/WO_3_. As shown in Fig. 1b and c, the AA degradation and the concurrent CO_2_ production over PA/WO_3_ were about 2.7 times and 4.0 times higher than that of Pt/WO_3_, respectively. It should be also noted that PA/WO_3_ exhibited similar enhanced PCD activities under different irradiation conditions of blue LED (*λ* = 460 nm), UV LED (*λ* = 365 nm) and halogen lamp (*λ* > 420 nm) (Supplementary Fig. 3, Supplementary Table 2). The AQE of PA/WO_3_ in the PCD of AA reached 16.1% and 22.3% under blue LED (*λ* = 460 nm) and UV LED (*λ* = 365 nm) irradiation, respectively, which were much higher than that of bare WO_3_ (Supplementary Fig. 5). PA/WO_3_ rapidly degraded AA of different initial concentrations ([AA]_0_ = 120–700 ppmv) under blue LED irradiation (Supplementary Fig. 6). As [AA]_0_ increased, AQE continued to increase until [AA]_0_ reached 600 ppmv. This indicates that more AA molecules are concentrated in the surface region with increasing the gas-phase AA concentration, implying the unique AA-accumulating behavior of PA/WO_3_. An AQE as high as 64.3% was achieved when [AA]_0_ was 700 ppmv. As far as we know, this AQE value is significantly higher than any other reported ones for the visible-light-driven PCD of AA (Supplementary Table 3). In addition, the AQE of PA/WO_3_ is also remarkable to our knowledge among the reported WO_3_-based visible-light PCD systems (Supplementary Table 4). To check the long-term durability of the PA component, the PA/WO_3_ sample that had been stored for 6 months under ambient conditions was tested for its PCD activity, which was little different from that of the fresh PA/WO_3_. This demonstrates that the PA/WO_3_ sample can be kept in long-term storage without losing its catalytic activity (see Supplementary Fig. 7). The above results showed the excellent performance of PA/WO_3_ as a visible-light photocatalyst for the degradation of AA that is a common indoor air pollutant.

To clarify the synergistic effect between PA and WO_3_, other iodine-containing inorganic compounds including NaIO_4_, NaIO_3_, NaI, and HIO_3_ were also tested for their effects on the PCD activity of WO_3_. Their activity decreased in the following order: PA/WO_3_ » NaIO_4_/WO_3_ > NaIO_3_/WO_3_ ≈ WO_3_ > HIO_3_/WO_3_ > NaI/WO_3_ (Fig. 1d and Supplementary Table 1). Only periodate compounds showed obvious enhancement effects on the PCD activity of WO_3_. In general, periodate-based compounds with high-valence-state iodine exhibit stronger electron-accepting and oxidizing capacity. The standard 2-electron reduction potential of IO_4_^−^ was much higher than that of IO_3_^−^ [*E*^0^ (IO_4_^−^/IO_3_^−^) = +1.623 V_NHE_)^35,36^ and (*E*^0^ (IO_3_^−^/HIO_2_) ≈ +0.88 V_NHE_]^37^. Therefore, periodate ions should be responsible for the efficient trapping of CB electrons in the combined WO_3_–IO_4_^−^ system. However, it is interesting to note that the PCD activity of NaIO_4_/WO_3_ (*k*_d_ = 9.65 × 10^−3^ min^−1^) was much lower than that of PA/WO_3_ (*k*_d_ = 39.3 × 10^−3^ min^−1^). We observed that the surface of the PA/WO_3_ catalyst became wet spontaneously after its exposure to ambient air while this phenomenon did not occur at all in the case of NaIO_4_/WO_3_. A similar effect of PA was also found on N-TiO_2_ (Supplementary Fig. 8). Based on this observation, we hypothesized that this spontaneously formed surface layer may influence the photocatalytic activity of PA/WO_3_ during the PCD of AA and further investigated the effect of water on the PCD activity.

**Fig. 2:**
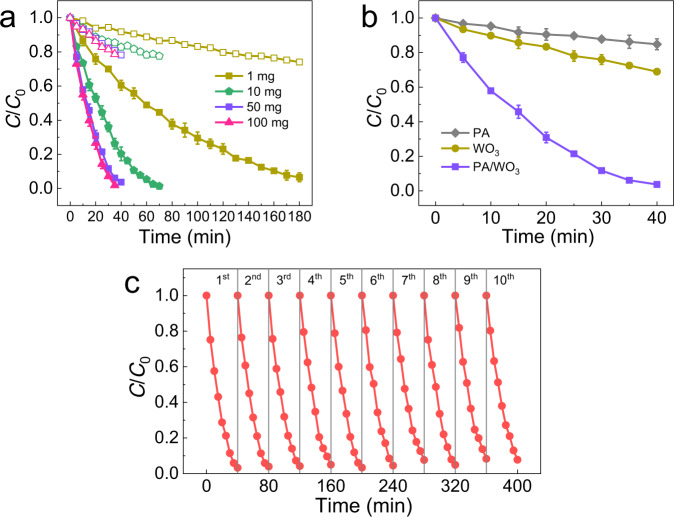
Visible light-driven PCD of formaldehyde (FA) at 500 ppbv on PA/WO_3_. **a** The dark control tests (open symbols) and the PCD tests (filled symbols) over PA/WO_3_ (1:1) with different catalyst mass (e.g., 10 mg catalyst composed of 5 mg PA and 5 mg WO_3_). **b** PCD of FA on PA/WO_3_, bare WO_3_ and PA. Error bars are defined as standard deviation. **c** Repeated PCD cycles of FA degradation over 50 mg PA/WO_3_. Experimental conditions: [FA]_0_ = 500 ppbv; blue LED (*λ* = 460 nm) intensity of 2.0 mW/cm^2^; RH 65%; reaction temperature of 30 °C.

The above PCD experiments employed high concentrations of AA ranging in 120–700 ppmv, which is unrealistically high for indoor environments. To demonstrate the performance of the PA/WO_3_ photocatalyst in a more realistic condition, the PCD of formaldehyde (FA) on PA/WO_3_ was additionally tested at a much lower concentration of 500 ppbv in a larger reactor (1.5 L) (compared with the PCD condition of AA) (see Fig. 2). FA is a common indoor air pollutant and a human carcinogen classified by the World Health Organization (WHO). The FA PCD tests were conducted under blue LED (*λ* = 460 nm) irradiation. After 30 min PCD reaction, the concentration of FA decreased from 500 to 58 ppbv (lower than the limit concentration allowed by WHO, 80 ppbv^38^) over 50 mg PA/WO_3_. Note that FA could be removed by PCD using as low as 1 mg PA/WO_3_. The activity of PA/WO_3_ was far higher than bare WO_3_ (Fig. 2b), and it remained active even after 10 PCD cycles (Fig. 2c). The above results confirm that PA/WO_3_ has an excellent capacity for degrading hydrophilic VOCs (FA and AA) selectively in wide-range concentrations (500 ppbv−700 ppmv). No other existing indoor air purification methods have such effective, durable, and selective capacity for the removal of indoor aldehydes under ambient conditions.

### Roles of PA and in situ formed water layer

**Fig. 3:**
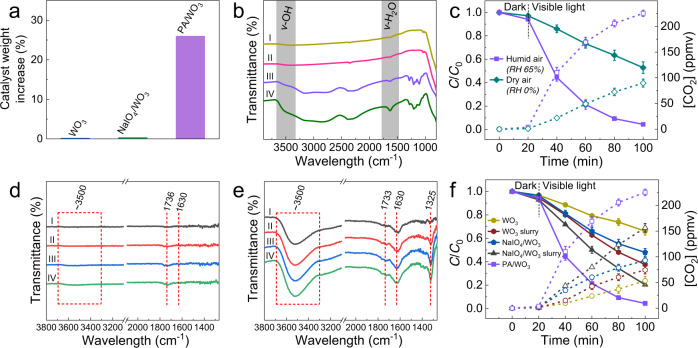
Formation and effects of the in situ formed water layer. **a** Catalyst weight increase of PA/WO_3_, NaIO_4_/WO_3_, and bare WO_3_ samples after exposing each catalyst under humid air (RH 65%) for 30 min. RH is defined as relative humidity. **b** FT-IR spectra of bare WO_3_ (I) before and (II) after exposure to humid air, and those of PA/WO_3_ (III) before and (IV) after exposure to humid air for 30 min. **c** PCD activity of PA/WO_3_ for acetaldehyde (AA) degradation after exposure to dry and humid air for 30 min. In situ DRIFT spectra for **d** PA/WO_3_ after exposing the sample to 300 ppmv AA/dry air stream and **e** PA/WO_3_ in 300 ppmv AA/RH 65% air stream for (I) 10 s, (II) 1 min, (III) 3 min, and (IV) 5 min. The spectra of the dry PA/WO_3_ surface were collected and used as the background. **f** PCD activity of bare WO_3_, NaIO_4_/WO_3_, and their slurries containing 26 wt% water for the degradation of AA. The dashed lines with open symbols represent the CO_2_ concentration generated from AA degradation. Error bars are defined as standard deviation. Experimental conditions: [AA]_0_ = 120 ppmv; visible light (*λ* > 420 nm) intensity of 2.2 mW/cm^2^; sample amount of 50 mg (with 13 mg of extra water in the case of WO_3_ slurry and NaIO_4_/WO_3_ slurry); reaction temperature of 30 °C.

PA molecules with hydroxyl groups are highly hygroscopic due to the hydrogen bonding between water molecules and the hydroxyl groups in PA^39^. On the other hand, NaIO_4_ is not hygroscopic due to the absence of hydroxyl groups. To evaluate the water absorption capacity, fresh air with 65% RH was flowed over the surface of bare WO_3_, NaIO_4_/WO_3_, and PA/WO_3_ samples for 30 min; then, the weight increased in each catalyst sample was measured (Fig. 3a). The weight of bare WO_3_ and NaIO_4_/WO_3_ was not changed while that of PA/WO_3_ increased by ~26%, which can be attributed to liquid water condensed on PA/WO_3_ from the humid air. Although water molecules should be adsorbed on the bare WO_3_ surface via hydrogen bonding with the surface hydroxyl groups^40^, the adsorbed water molecules do not induce the condensation of the water layer on WO_3_. It seems that the presence of PA facilitates the condensation of the water layer on the surface of the catalyst. To further confirm the in situ formation of the water layer via condensation, the PA/WO_3_ coated on the glass was exposed in ambient air for 10 min, then the sample was in situ observed with microphotography (see the Supplementary video). We observed that the formation of condensing water layer induces the movement of WO_3_ particles. When the wet PA/WO_3_ sample was dried by an external heater, PA crystals appeared with ceasing the moving of WO_3_ particles. Upon stopping the heating, the water absorption process resumed with redissolving PA crystals and moving WO_3_ particles again. The FT-IR spectral changes in PA/WO_3_ and bare WO_3_ before and after the exposure to humid air also show such a trend clearly (Fig. 3b). The peak assigned to the stretching vibration of surface OH is located at 3600−3200 cm^−1^ (ν-OH), while the signal of bending vibration of adsorbed water appeared at ~1630 cm^−1^ (ν-H_2_O)^41^. The signals of both (ν-OH) and (ν-H_2_O) for bare WO_3_ remained almost unchanged after exposure to humid air, which confirms that bare WO_3_ surface does not induce significant water adsorption. However, these signals on PA/WO_3_ were markedly enhanced after exposure to humid air, which indicates the condensation of water. The strong hydrogen bonding between the hydroxyl groups of PA and water molecules should make the condensation of water vapor highly exothermic at ambient conditions, where the negative ▵*H* outweighs the entropy decrease of water vapor condensation to make the overall condensation process thermodynamically spontaneous (▵*G* < 0). Therefore, the interfacial characteristics in the catalyst surface region of PA/WO_3_ should be changed from the two-phase gas–solid interaction to the three-phase gas–liquid–solid interaction after the PA-induced condensation of the water layer.

To clarify the effect of water, the PCD activities of PA/WO_3_ were compared between the dry (0% RH) and humid air (65% RH) conditions (Fig. 3c). Both the degradation of AA and the accompanying production of CO_2_ over PA/WO_3_ were significantly reduced under the dry air condition, which confirms that the role of water vapor is critical in controlling the overall PCD process. However, if not in the dry condition, the humidity variation ranging in RH 40–90% has a minor influence on the PCD of AA on PA/WO_3_ (see Supplementary Fig. 9). It should be also noted that PA/WO_3_ showed higher PCD activity than bare WO_3_ even in dry air (Figs. 1a vs. 3c). The adsorption of VOCs is the first step in gas–solid photocatalytic reaction, and the adsorption properties of VOCs often significantly affect the PCD efficiency^42^. In the case of PA/WO_3_ in humid air condition, the spontaneous formation of the liquid water layer on the catalyst surface should hinder the direct contact between VOC molecules and the WO_3_ surface, which makes the reaction medium involve all three phases. As a result, the first step in PCD should be changed from the adsorption of AA on the WO_3_ surface to the dissolution of AA in the surface water layer. Hydrophilic AA is highly soluble in water at room temperature^43^. The dark adsorptive removal of AA over PA/WO_3_ was compared after 30 min equilibration under dry air and humid air (65% RH). It is found that 6.5% and 18.6% of AA is adsorbed on the catalyst surface within 100 min under dry and humid air conditions, respectively, which indicates that the existence of a water layer promoted the uptake of AA on the catalyst surface.

The relation between AA and the surface water layer was further investigated by in situ DRIFT spectroscopy under dark conditions. When exposing PA/WO_3_ to 300 ppmv AA/dry airflow (see Fig. 3d), no peaks corresponding to ν-OH (3600−3200 cm^−1^) and ν-H_2_O (~1630 cm^−1^) were found, which indicates that the formation of the surface water layer is negligible. The small peak located at 1736 cm^−1^ (ν-C = O vibration mode in aldehydes) is assigned to AA adsorbed via hydrogen bonding with a surface OH group^44^. On the other hand, the ν-OH (3600−3200 cm^−1^), ν-H_2_O (~1630 cm^−1^), and ν-C = O (1733 cm^−1^) peaks all increased significantly when exposing PA/WO_3_ to 300 ppmv AA/65% RH airflow, which indicates the formation of the surface water layer and the enrichment of AA (see Fig. 3e). Moreover, a distinct peak corresponding to C–O stretching vibration (ν-C–O) for carboxylic acids (1325 cm^−1^)^45,46^ gradually appeared with time. This implies that the in situ water layer formation facilitates not only adsorption/dissolution of AA but also partial pre-oxidation of AA, which subsequently accelerates the PCD process under irradiation.

To further confirm the role of the surface water layer, an aliquot of water was added to WO_3_ powder to make a slurry (with and without NaIO_4_): 13 mg water was well mixed with 50 mg WO_3_ or 50 mg NaIO_4_/WO_3_ (w/w: 1/1) to form slurries. The resulting slurry photocatalysts and the corresponding dry photocatalysts were compared for the PCD of AA (see Fig. 3f). The slurry photocatalysts (with and without NaIO_4_) showed higher PCD activity than the corresponding dry samples and the NaIO_4_/WO_3_ slurry exhibited higher PCD activity than pure WO_3_ in either slurry or dry state. This confirms that the high activity of PA/WO_3_ should be ascribed to the combined action of IO_4_^−^ and water. The PA acidity in PA/WO_3_ may play a role as well since the pH of the PA solution was 1.5 whereas that of NaIO_4_ solution was 4.6. To test the acidity effect, the pH of NaIO_4_ solution was adjusted to 1.5 with adding iodic acid when preparing NaIO_4_/WO_3_ and the PCD activity of the acidified NaIO_4_/WO_3_ slurry was much higher than that of NaIO_4_/WO_3_ slurry (see Supplementary Fig. 10). Considering that iodic acid alone did not promote the PCD activity of WO_3_, the above result implies that the PA acidity in the water layer should contribute to the high PCD activity of PA/WO_3_. This might be related to the fact that the reduction of periodate is favored at an acidic condition (IO_4_^−^ + 2H^+^ + 2e^−^ → IO_3_^−^ + H_2_O). Therefore, the water-rich surface over PA/WO_3_ should scavenge photogenerated holes more efficiently to produce •OH and AA molecules that are dissolved and concentrated within the thin water layer should undergo the subsequent PCD reactions in the solid–water interface. This process should be much faster than that in the traditional gas–solid interfacial PCD reaction since the AA molecules in the surface water layer is far more concentrated and oxidized than those in the gas phase. The synergy between IO_4_^−^ and the water layer is further discussed in the following section.

**Fig. 4:**
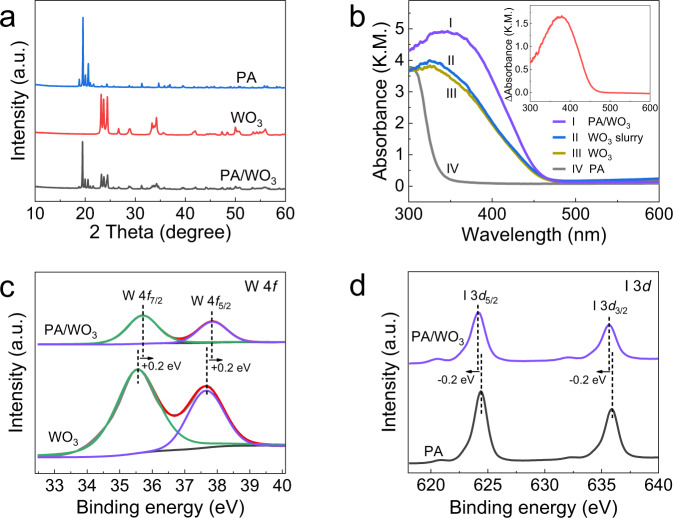
Characterizations of PA, WO_3_, and PA/WO_3_. **a** X-ray diffraction spectra. **b** Diffuse reflectance UV–visible absorption spectra (DRS) (Inset is the difference DRS spectrum which is obtained from the spectrum I minus the spectrum III). XPS spectra of **c** W 4*f* and **d** I 3*d* band.

The structural and surface properties of PA-treated WO_3_ were evaluated by using XRD, FE-SEM, N_2_ adsorption–desorption, XPS, DRS techniques, and density functional theory (DFT) calculations. As shown in Fig. 4a, the position of diffraction peaks in the XRD pattern of PA/WO_3_ was consistent with those of monoclinic WO_3_ (JCPDS-ICDD card #75-2072) and HIO_4_·2H_2_O (JCPDS-ICDD card #74-0334). No characteristic peaks of any other phases were observed, indicating that the original crystal form of WO_3_ and PA was not affected in the prepared PA/WO_3_ sample. In addition, PA treatment had a negligible effect on the BET surface area (*S*_BET_), pore structure, and morphology of WO_3_ (see Supplementary Figs. 11 and 12). To evaluate the light absorption capacity of samples, the UV–vis spectra of WO_3_ and PA/WO_3_ are compared in Fig. 4b. The light absorption capacity of WO_3_ was clearly enhanced in the range of 300–450 nm after combining with PA. This PA-enhanced absorption is more clearly seen in the difference DRS spectra (see the inset of Fig. 4b). The extra light absorption should not be ascribed to the formation of an in situ water layer on PA/WO_3_ since the absorption spectrum of WO_3_ slurry (26 wt% water content) was a little different from that of dry WO_3_ powder. Therefore, the enhanced absorption in the 300–450 nm region (inset of Fig. 4b) where PA itself is optically transparent, should be ascribed to the surface interaction between PA and WO_3_. The XPS analysis was also employed to investigate the interaction between PA and WO_3_. In the measured W 4*f* XPS spectra (Fig. 4c), the bare WO_3_ sample shows two binding energy (BE) peaks at 35.5 and 37.7 eV, which are assigned to 4*f*_7/2_ and 4*f*_5/2_ of W^6+^, respectively^47^. The two peaks of PA/WO_3_ were shifted by ~0.2 eV to higher BE in comparison to those of bare WO_3_. Several studies showed that the inorganic acid treatment of TiO_2_ shifts the metal BE higher because of the strong interaction between the acid anions and metal cations^48–50^. It can be reasonably inferred that the oxygen atom in the PA molecule could be directly complexed with W cation on the surface of WO_3_ in the form of “W–O–I–(OH)_*n*_”. In this complex, the electron density on the W cation should be further decreased by the strong electron-withdrawing character of the iodine cation (I^7+^), leading to the slight increase in the BE of W 4*f*. The I 3*d* XPS spectra (shown in Fig. 4d) may further support such a conclusion. The BEs of I 3*d*_5/2_ and I 3*d*_3/2_ for PA are at 624.4 and 635.9 eV, respectively, but those of PA/WO_3_ exhibit a negative shift by ~0.2 eV, which indicates the increased electron density on iodine. This suggests that the charge transfer in the “W–O–I–(OH)_*n*_” complex is induced by the “electron-withdrawing effect” of the iodine cation to decrease the electron density on W but to increase that on I. Such charge transfer complex formation on the WO_3_ surface not only facilitates the separation of photogenerated electron–hole pairs but also promotes the light absorption ability of WO_3_^51–53^, as supported by the enhanced light absorption of PA/WO_3_ in Fig. 4b. Moreover, the DFT calculation that was carried out to investigate the interaction between PA and WO_3_ surface shows that the calculated adsorption/binding energy of PA on WO_3_ surface is −3.7 eV, a high value which usually implies the formation of strong chemical bonds^54^. The charge density difference analysis shows that there is an electron-depleted region on the WO_3_ surface and an electron-gaining region around the iodine center. This clearly indicates that the charge is transferred from the WO_3_ surface to PA, which is consistent with the XPS results (see Supplementary Fig. 13).

**Fig. 5:**
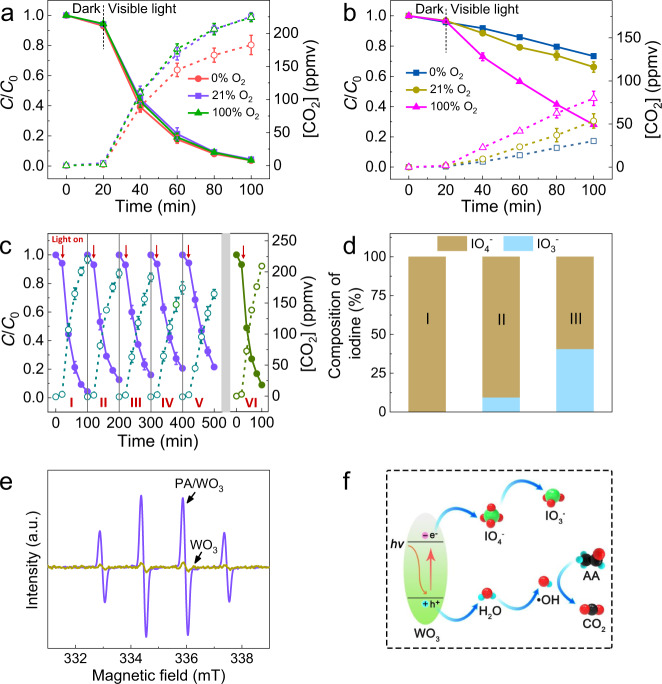
Effects of O_2_ and PA. Effect of O_2_ concentration on the PCD of acetaldehyde (AA) over **a** PA/WO_3_ and **b** bare WO_3_. **c** Repeated PCD cycles of AA degradation and the concurrent production of CO_2_ over PA/WO_3_ (I, 1st cycle; II, 2nd cycle; III, 3rd cycle; IV, 4th cycle; V, 5th cycle), (VI) PA/WO_3_ was regenerated after 5th cycle. Error bars are defined as standard deviation. **d** Relative distribution of iodine species in (I) fresh PA/WO_3_, PA/WO_3_ used after (II) 1st PCD cycle and (III) 5th PCD cycle of AA degradation in **c**: IO_4_^−^ (brown) and IO_3_^−^ (cyan). **e** EPR spectra probing the photogeneration of •OH adduct (•OH-DMPO) in an aqueous catalyst slurry containing DMPO (*C*_0_ = 10 mM). **f** Proposed PCD mechanism of AA. The dashed lines with open symbols represent the CO_2_ concentration generated from AA degradation. Experimental conditions: [AA]_0_ = 120 ppmv; visible light (*λ* > 420 nm) intensity of 2.2 mW/cm^2^; sample amount of 50 mg; RH 65%; reaction temperature of 30 °C.

The role of dioxygen was also investigated. The PCDs of AA over PA/WO_3_ and bare WO_3_ were conducted under different O_2_ concentrations (Fig. 5a and b). In general, the PCD rate is faster with producing more CO_2_ at higher O_2_ concentration (as shown in Fig. 5b) since dioxygen serves as the main electron acceptor in gas–solid interface PCD reaction with the concurrent formation of reactive oxygen species (e.g., O_2_•^−^, HO_2_•)^55^. This effectively transfers electrons to facilitate the charge carrier separation, and the resulting reactive oxygen species play a key role in oxidizing VOCs. However, the PCD of AA over PA/WO_3_ was a little dependent on O_2_ concentration and 120 ppmv of AA could be completely removed even in the absence of O_2_ although the concurrent production of CO_2_ was a little reduced without O_2_ (Fig. 5a). This indicates that PA is a much stronger electron acceptor than dioxygen [*E*^0^ (IO_4_^−^/IO_3_^−^) = +1.623 V_NHE_, *E*^0^ (O_2_/O_2_•^−^) = −0.33 V_NHE_, *E*^0^ (O_2_/HO_2_•) = −0.05 V_NHE_]^13,35,36^. The presence of O_2_ did not enhance the degradation rate of AA but moderately increased CO_2_ generation. This is consistent with the fact that O_2_ is needed as a reagent for the complete mineralization of aldehydes into CO_2_^56^.

The role of PA as the main electron acceptor implies that it should be consumed with irradiation time. Multi-cycle PCD experiments of AA over PA/WO_3_ were conducted (Fig. 5c), which indeed exhibited a gradual decrease in the PCD rate. However, the PCD activity was maintained relatively high for a long irradiation period. In a comparison of the first and the fifth cycles, the removal of AA in 80 min decreased from 95.6% to 78.5%, and the fraction of mineralized AA (estimated from the CO_2_ production) in 80 min was reduced from 93.9% to 70.3%. Even after the fifth cycle, the PCD activity was far higher than bare WO_3_ (Fig. 1). FE-SEM, TEM, and XPS results showed that the morphology and surface chemical state of WO_3_ in PA/WO_3_ were little changed after the PCD reaction of AA (see Supplementary Figs. 12 and 14). The analysis of the iodine-containing species in PA/WO_3_ during the cyclic experiments (Fig. 5d) found that the IO_4_^−^ portion decreased from 100% in the fresh PA/WO_3_ to 90.5% after the first cycle, and further to 59.3% after the fifth cycle of AA degradation. At the same time, the IO_3_^−^ portion increased from 0% to 9.5% after the first cycle, and further to 40.7% after the fifth cycle. On the other hand, no I^−^ was detected throughout the multicycle experiments. It seems that the photocatalytic reaction of IO_4_^−^ with CB electrons leads to the production of IO_3_^−^ as a main product on the visible light-irradiated PA/WO_3_. The possible reoxidation of IO_3_^−^ to IO_4_^−^ can be ruled out since the control PCD tests of AA using NaIO_3_/WO_3_ and HIO_3_/WO_3_ showed no production of IO_4_^−^. Therefore, the gradual decline of catalyst activity of PA/WO_3_ in Fig. 5c should be ascribed to the consumption of IO_4_^−^. To regenerate the photocatalyst, the used PA/WO_3_ was washed with water and reloaded with 0.11 mol/L PA solution by following the same procedure as in the preparation of the fresh PA/WO_3_. The regenerated PA/WO_3_ catalyst almost fully recovered its original activity (see Fig. 5c, VI), which confirms the critical role of PA as an electron acceptor in the PCD of AA.

On the other hand, as for the hole transfer part, the photocatalytic production of OH radicals is one of the primary mechanisms by which photocatalysts degrade VOCs. The ability of PA/WO_3_ to produce •OH was tested using a chemical trapping method using DMPO as a spin trap reagent^57^. After 10 min of blue LED irradiation (460 nm, 2.0 mW/cm^2^), EPR signals for the •OH-DMPO adduct appeared as shown in Fig. 5e. Note that PA/WO_3_ produced a prominent signal whereas bare WO_3_ generated an insignificant one. In general, bare WO_3_ is not an efficient photocatalyst because of the lower CB edge (~0.4 V_NHE_)^58^ that does not provide a sufficient potential to reduce O_2_ [*E*^0^ (O_2_/O_2_•^−^) = −0.33 V_NHE_ and *E*^0^ (O_2_/HO_2_•) = −0.05 V_NHE_]^13,20^. The inability of O_2_ to scavenge CB electrons in WO_3_ results in fast recombination and lower photocatalytic activity. After introducing PA, the periodate ions in the in situ formed surface water layer can scavenge CB electrons, which subsequently retards the charge recombination and makes more holes available for the production of •OH that should be the primary oxidant in the PCD process. The photocatalytic oxidation mechanism (see Fig. 5f) can be briefly proposed as follows (Eqs. (1)–(5)):

1 $${{{{{{\rm{WO}}}}}}}_{3}+{{\rm {h}}}{\upnu }\to {{{\rm {h}}}}_{{{{{{\rm{vb}}}}}}}^{+}+{{{{{{\rm{e}}}}}}}_{{{{{{\rm{cb}}}}}}}^{-}$$

2 $${{{{{{\rm{HIO}}}}}}}_{4}({{{{{\rm{PA}}}}}})\to {{{{{{\rm{H}}}}}}}^{+}+{{{{{{{\rm{IO}}}}}}}_{4}}^{-}({{{{{\rm{in}}}}}}\,{{{in}}}\, {{{situ}}}\,{{{water}}}\,{{{layer}}})$$

3 $${{{{{{\rm{IO}}}}}}}_{4}^{-}+2{{{{{{\rm{H}}}}}}}^{+}+2{{{{{{\rm{e}}}}}}}_{{{{{{\rm{cb}}}}}}}^{-}\to {{{{{{\rm{IO}}}}}}}_{3}^{-}+{{{{{{\rm{H}}}}}}}_{2}{{{{{\rm{O}}}}}}$$

4 $${{{{{{\rm{H}}}}}}}_{2}{{{{{\rm{O}}}}}}+{{{{{{\rm{h}}}}}}}^{+}\to \bullet {{{{{\rm{OH}}}}}}+{{{{{{\rm{H}}}}}}}^{+}$$

5 $${{{{{\rm{AA}}}}}}+\bullet {{{{{\rm{OH}}}}}}+{{{{{{\rm{O}}}}}}}_{2}\to\to {{{{{{\rm{CO}}}}}}}_{2}$$

### Selective PCDs for hydrophilic vs. hydrophobic VOCs

**Fig. 6:**
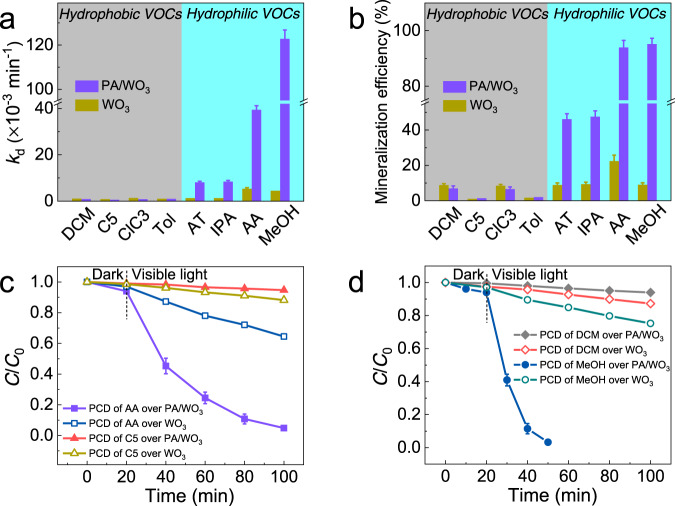
Selective removal of hydrophilic volatile organic compounds (VOCs) over PA/WO_3_. **a** PCD rate constants (*k*_d_) and **b** the mineralization efficiencies of different VOCs over bare WO_3_ and PA/WO_3_. PCD of the mixture of **c** AA and C5 and **d** MeOH and DCM. Error bars are defined as standard deviation. Experimental conditions: [VOC]_0_ = 120 ppmv; visible light (*λ* > 420 nm) intensity of 2.2 mW/cm^2^; sample amount of 50 mg; RH 65%; reaction temperature of 30 °C. AA acetaldehyde, IPA isopropanol, AT acetone, MeOH methanol, DCM dichloromethane, C5 *n*-pentane, ClC_3_ *n*-chloropropane, Tol toluene.

Other VOCs including methanol (MeOH), isopropanol (IPA), acetone (AT), *n*-pentane (C5), dichloromethane (DCM), *n*-chloropropane (ClC_3_), and toluene (Tol) were also tested for their PCDs by bare WO_3_ and PA/WO_3_. As shown in Fig. 6a and b, the PCD behaviors are clearly different depending on the kind of target VOCs. Compared with bare WO_3_, PA/WO_3_ increased the PCD rate constant (*k*_d_) by 6.7, 6.5, and 7.5 times for the degradation of IPA, AT, and AA, respectively. Accordingly, the mineralization efficiency of IPA, AT, and AA over PA/WO_3_ was 5.2, 5.2, 4.2 times higher than that over bare WO_3_, respectively. Note that the PCD of MeOH was even more dramatically enhanced than that of AA on PA/WO_3_: *k*_d_ for MeOH on PA/WO_3_ (122.71 × 10^−3^ min^−1^) was 27.8 times higher than that of bare WO_3_ (*k*_d_ = 4.42 × 10^−3^ min^−1^). The PCDs of all the hydrophilic VOC molecules were markedly enhanced on PA/WO_3_, which implies that they can be easily dissolved and concentrated in the in situ formed surface water layer to facilitate their PCD. However, the situation was markedly different for the PCDs of hydrophobic VOC molecules. The PCD rates of DCM, C5, Tol, and ClC_3_ were very low over bare WO_3_ (*k*_d_ < 2.0 × 10^−3^ min^−1^) and the corresponding mineralization efficiencies of these VOCs were lower than 10%. Unlike the case of hydrophilic VOCs which exhibited the dramatic PA-enhanced effect, PA/WO_3_ slightly hindered the PCDs of hydrophobic VOCs, compared with bare WO_3_. The water layer on PA/WO_3_ should not dissolve hydrophobic VOC molecules and their PCDs should be limited under such conditions. The markedly contrasting PCD behaviors of PA/WO_3_ between hydrophobic and hydrophilic VOCs should be ascribed to their different solubility in the in situ formed surface water layer.

The selective PCDs in VOC mixtures were also investigated under visible-light irradiation (*λ* > 420 nm), as shown in Fig. 6c and d. With PA/WO_3_, more than 95% of AA could be removed in the AA/C5 mixture after 80 min of PCD while the concentration of C5 was almost unchanged. In the same manner, only MeOH could be selectively removed in MeOH/DCM mixture. On the other hand, PCD over bare WO_3_ removed 35.5% of AA and 11.8% of C5 in AA/C5 mixture in 80 min; 24.7% of MeOH and 12.8% of DCM in MeOH/DCM mixture. This clearly shows that PA/WO_3_ has good selectivity for the degradation of hydrophilic VOCs while bare WO_3_ photocatalyst has much lower selectivity. It is quite clear that the presence of an in situ formed water layer on PA/WO_3_ greatly facilitates the selective removal of hydrophilic VOC molecules while keeping hydrophobic VOCs intact in the hydrophilic–hydrophobic VOC mixture. As a result, the highly enhanced and selective removal of hydrophilic VOCs is enabled by the PA-treatment of WO_3_ photocatalyst which induces the in situ formation of the water layer on the catalyst surface. The role of in situ water layer formation on the PCDs of VOCs has not been previously recognized and needs to be further investigated for the selectivity control of VOCs degradation.

## Discussion

The photocatalytic oxidation processes have been extensively investigated for the purification of polluted air and water. A variety of photocatalyst modification methods have been proposed and tested to improve the performance of PCD while many methods need complicated preparation procedures or costly materials such as noble metals. In this work, a very simple and inexpensive method of photocatalyst modification is proposed and demonstrated for the highly enhanced and selective degradation of VOCs. PA coating on photocatalysts proved to be an effective method to greatly enhance the photocatalytic activity of WO_3_, BiVO_4_, and N-TiO_2_ for the degradation of AA (a common indoor air pollutant) under visible light. In particular, PA/WO_3_ exhibited excellent performance in PCD of various hydrophilic VOCs. Under visible-light irradiation (*λ* > 420 nm), the PCD rate constant (*k*_d_) of IPA, AT, AA, and MeOH over PA/WO_3_ was 6.7, 6.5, 7.5, and 27.8 times higher than that of bare WO_3_. The hygroscopic property of PA enables the photocatalyst to form in situ surface water (self-wetting) layer in ambient air while the periodate ions in the in situ water layer serve as efficient electron acceptors on WO_3_ under visible light. In addition, PA formed surface complexes on WO_3_ surface not only to enhance the visible light absorption capacity but also to suppress the charge pair recombination by making PA scavenge CB electrons more efficiently. The multiple roles of PA induce a water layer on the photocatalyst surface, dissolve and concentrate hydrophilic VOCs in the water layer, and make more holes available to produce OH radicals. As a result, PA/WO_3_ photocatalyst is even more active than Pt-loaded WO_3_ (a popular but expensive visible-light active photocatalyst) for the degradation of AA.

PA is proposed as a low-cost component to replace costly Pt cocatalyst for the removal of hydrophilic VOCs. PA is nonvolatile and comparatively nontoxic and its release into the air can be safely neglected. AA degraded by PA/WO_3_ photocatalyst generated no detectable volatile byproducts. To check the possible generation of volatile iodine species (e.g., I_2_, HOI) from the transformation of PA during the PCD of AA on PA/WO_3_, the treated air was absorbed by phenol solution (1 mM) and analyzed for iodophenol (a product that should be generated from the reaction with volatile iodine species) by HPLC^59^. No iodophenol was detected, which indicated that no volatile iodine species was present in the treated air. No other gaseous organic products (e.g., FA, acetic acid) were found during the PCD of AA, which indicates the rapid mineralization of AA. The presence of a surface water layer can trap any hydrophilic intermediates and degrade them within the water layer without emitting them into the air phase. This self-wetting tri-phasic (air/water/catalyst) photocatalytic system facilitates the complete degradation of hydrophilic VOCs by providing the in situ water layer where the hydrophilic intermediates/byproducts are more efficiently retained and degraded in the aqueous phase whereas the common biphasic photocatalysis (air/catalyst) often generates gaseous intermediates.

From a practical point of view, a notable advantage is that the uptake of water vapor onto PA/WO_3_ and the subsequent drying are reversible (as shown in the Supplementary video). This makes the wetting and drying process repeated depending on the humidity condition of the ambient air. As wetting a large surface is not convenient for practical applications, it is suggested that PA/WO_3_ be employed as a replaceable filter component in air purifiers (especially for the removal of aldehydes such as AA and FA), not to be coated over the support with a large surface area. The reaction stoichiometry indicates that 5 moles of PA are needed to degrade 1 mole of AA (CH_3_CHO + 5IO_4_^−^ → 2CO_2_ + 5IO_3_^−^). The cost of PA replacement should not be a problem because of its low price (99.9% purity, ~10.5$/kg in the USA). Our analysis showed that 40.7% of initial PA (25 mg PA in 50 mg PA/WO_3_) was reduced to iodate along with mineralizing 0.29 mg AA during five PCD cycles of AA (see Fig. 5d). This corresponds to the consumption of 1 mole PA for the degradation of 0.15 mole AA, which is close to the theoretical value (5:1). Based on this ratio, we estimate that 1 g PA is consumed to purify 317 m^3^ of indoor air contaminated with [AA] = 90 μg/m^3^ (10 times higher than the US EPA reference concentration for chronic inhalation exposure^60^, 9 μg/m^3^). Moreover, the used filter can be washed with water and regenerated by replenishing PA or by recycling iodate back to periodate via electrochemical^61^ or other economical chemical oxidation methods. Alternatively, a photoelectrochemical (PEC) filter device that can regenerate in situ periodate as soon as it is converted into iodate under irradiation in the PA/WO_3_ filter plans to be developed in a further study. To further investigate the effect of the geometric surface area of the coated photocatalyst, the PCD activity (for removing 500 ppbv FA in 1.5 L air) of PA/WO_3_ was compared between the different geometric coating areas of 1 cm^2^ vs. 4 cm^2^ (see Supplementary Fig. 15). The PCD activity little decreased with decreasing the catalyst coating area from 4 to 1 cm^2^, which shows that the ratio of treated air volume to catalyst coating area can reach over 1.5 L/cm^2^. This clearly demonstrates that PA/WO_3_ has a good capacity in purifying large volumes of air. In practical applications, this ratio is expected to be further enhanced by optimizing the catalyst dosage and coating thickness. Specific design parameters (e.g., airflow rate, catalyst dosage, thickness and area of catalyst coating, etc.) need to be carefully adjusted according to the actual application purpose. These engineering parameters remain to be investigated in future works. It is worth noting that the PA/WO_3_ photocatalyst demonstrated good applicability for a wide range of VOC concentrations (500 ppbv−700 ppmv).

**Fig. 7:**
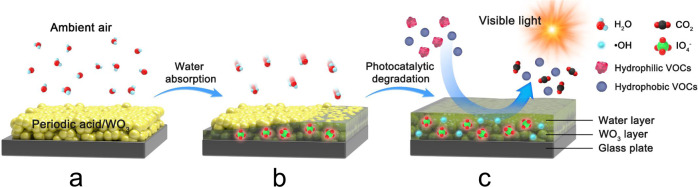
Schematic illustration of the working mechanism of PA/WO_3_ photocatalysis for the selective PCD. **a** WO_3_ is coated with periodic acid (PA) to form PA/WO_3_. **b** In situ surface water layer is formed due to the hygroscopic property of PA in ambient air while the periodate ions (IO_4_^−^) in the in situ water layer serve as efficient electron acceptors. **c** Under visible light, the photogenerated electrons are rapidly scavenged by IO_4_^−^ while holes produce •OH radicals which subsequently degrade hydrophilic VOCs dissolved into the in situ water layer. However, hydrophobic VOCs remain intact during photocatalysis due to the “water barrier effect”.

On the other hand, the inability of PA/WO_3_ to remove hydrophobic VOCs (e.g., DCM, C5, ClC_3_, and Tol) is a serious limitation for general purpose applications. However, its ability to degrade hydrophilic VOCs selectively against hydrophobic VOCs can be exploited in a specific application (see Fig. 7). In addition, the in situ formed water layer can protect the photocatalyst surface from fouling with inhibiting the deposition of hydrophobic components (e.g., indoor particulate matters like cooking particles, various VOCs containing halogen/phosphorus/silicon found in indoor environments)^62–64^. As a viable strategy for exploiting in situ water layer formation in ambient air photocatalysis, the PA/photocatalyst may be combined in a hybrid air treatment system where hydrophilic VOCs (that can be rapidly degraded by photocatalysis) are selectively degraded by the PA/photocatalyst and then hydrophobic VOCs (that are more recalcitrant against photocatalysis) are removed by other methods such as adsorption and thermal catalysis. Such an approach combines the advantages of various technologies to develop a more efficient and economical method of controlling VOCs.

## Methods

### Materials

The chemicals used in this study are as follows: Tungsten oxide (WO_3_, nanopowder, Sigma-Aldrich), titanium dioxide (TiO_2_, P25, nanopowder, Evonik), bismuth vanadate (BiVO_4_, nanopowder, Alfa Aesar), PA (HIO_4_·2H_2_O, ≥99.0%, Sigma-Aldrich), iodic acid (HIO_3_, ≥99.5%, Sigma-Aldrich), sodium periodate (NaIO_4_, ≥99.8%, Sigma-Aldrich), sodium iodate (NaIO_3_, 99%, Sigma-Aldrich), sodium iodide (NaI, ≥99.0%, Sigma-Aldrich), chloroplatinic acid (H_2_PtCl_6_·*x*H_2_O, ≥99.9%, Sigma-Aldrich), MeOH (CH_3_OH, 99.9%, Samchun Chemicals), IPA ((CH_3_)_2_CHOH, 99.5%, Sigma-Aldrich), AT (CH_3_COCH_3_, 99.98%, Burdick Jackson), DCM (CH_2_Cl_2_, ≥99.8%, Sigma-Aldrich), C5 (CH_3_(CH_2_)_3_CH_3_, ≥99.0, Sigma-Aldrich), ClC_3_ (CH_3_CH_2_CH_2_Cl, 99%, Alfa Aesar), 5,5-dimethyl-1-pyrroline-*N*-oxide (DMPO, ≥ 98.0, Sigma-Aldrich). Tol (300 ppmv, N_2_ balance), AA (1000 ppmv, N_2_ balance), FA (100 ppmv, N_2_ balance), high-purity synthetic air (79% N_2_/21% O_2_) were purchased from Deokyang Company. All chemicals were of reagent grade and used as received without further purification. Ultrapure deionized water (18 MΩ cm) prepared using a Millipore system was used.

### Preparation of photocatalyst materials

Commercial WO_3_ powder sample was treated with PA. Typically, 0.25 g WO_3_ was dispersed in 10 mL 0.11 mol/L PA solution under sonication. The mixture was stirred at room temperature for 12 h, and the obtained suspension was completely dried in an oven at 80 °C. To compare with PA/WO_3_ sample, control WO_3_ samples that were treated using NaIO_4_, NaIO_3_, HIO_3_, or NaI as an alternative reagent were also prepared under the same experimental conditions.

Two other visible light photocatalysts of N-doped TiO_2_ (N-TiO_2_) and BiVO_4_ were also treated with PA and compared with WO_3_ for the effects of PA treatment. N-TiO_2_ was prepared by high-temperature nitridation of TiO_2_^65^: commercial P25 powder was treated in a tubular furnace at 500 °C under NH_3_ gas flow (150 mL/min) for 5 h. Then, 0.25 g of N-TiO_2_ and BiVO_4_ were treated with 0.11 mol/L PA solution under the same condition as that of WO_3_. In addition, Pt/WO_3_ was prepared using a photodeposition method as another standard visible light photocatalyst^23,27^. Typically, an aqueous suspension of WO_3_ (0.5 g/L) was irradiated with a 200 W mercury lamp for 30 min in the presence of chloroplatinic acid as a Pt precursor and MeOH (1 mol/L) as an electron donor. The amount of platinum loading was fixed at 1 wt% for WO_3_. The resulting Pt/WO_3_ powder was collected by filtration and washed with deionized water.

### PCD experiments

The PCD of VOCs was conducted under visible light irradiation from a mercury lamp (custom-made), which was filtered through a 420-nm cutoff (*λ* > 420 nm) filter. The filtered light intensity on the photocatalyst was measured to be 2.2 mW/cm^2^ by a power meter (Newport 1918-R). A closed-circulation glass reactor (300 mL) with a quartz window (a radius of 3 cm) was used (see Supplementary Fig. 1). A magnetic bar was placed at the bottom of the glass reactor to stir the air. The reactor was connected to a gas chromatograph (GC-Agilent 6890 Plus) equipped with a methane converter, a Porapak R column, an automatic sampling valve using an air actuator, and a flame ionization detector. The relative humidity (RH) was adjusted to ~65% by bubbling air through a stainless-steel bottle containing deionized water. A heating device was used to maintain the temperature of the photocatalytic reactor at ~30 °C. To test the airtightness of the reactor, it was filled with high-purity synthetic air (79% N_2_/21% O_2_) and exposed to ambient air. A negligible increase in CO_2_ (<4 ppmv within 60 min) was detected, indicating good airtightness of the reactor system.

Photocatalyst powder (50 mg) was dispersed in water in a quartz glass sink which possessed a 2 cm × 2 cm groove to hold the catalyst component. The catalyst slurry was completely dried and placed in the reactor for PCD tests. Before each PCD experiment, the glass reactor was purged with high-purity synthetic air (79% N_2_/21% O_2_) and irradiated under the mercury lamp or LED lamp to degrade any organic impurities remaining on the photocatalyst surface. The cleaning irradiation continued until the photogeneration of CO_2_ was not detected.

Target VOCs tested in this study include FA, AA, IPA, AT, MeOH, DCM, C5, ClC_3_, and Tol. AA or Tol was introduced into the photocatalytic reactor through diluting the standard gas (1000 ppmv AA, 300 ppmv Tol in N_2_). For other VOCs (IPA, AT, MeOH, DCM, C5, or ClC_3_), a calculated amount of each liquid sample was injected into the reactor and subsequently vaporized into the gas phase. The initial concentration of VOCs was adjusted to 120 ppmv. After 20 min equilibration for complete dispersion and pre-adsorption of VOCs on the photocatalyst surface, the mercury lamp was turned on to initiate the PCD process. The removal of each VOC and the accompanying CO_2_ production was monitored in real-time by using a GC. All the control experiments were conducted under the same condition.

The PCD experiments of FA were carried out using a bigger reactor (1.5 L) instead of a 300 mL reactor to demonstrate the photocatalytic air treatment on a larger scale. A 460 nm-emitting LED (Luna Fiber Optic Korea, ICN14D-096) was employed as a light source. Photocatalyst powder (1, 10, 50, and 100 mg) was dispersed in water in a quartz glass groove (2 cm × 2 cm), which was dried and placed in the PCD reactor. Before each PCD test, the glass reactor was purged with high-purity synthetic air (79% N_2_/21% O_2_) and irradiated under an LED lamp to clean the photocatalyst surface. FA was introduced into the reactor through a mass flow controller regulating the standard gas (100 ppmv FA in N_2_) flow. The initial concentration of FA was adjusted at 500 ppbv, which is much lower than that of other VOCs (120 ppmv). This represents a more realistic test condition where FA is present in an indoor air environment. A photoacoustic gas monitor (LumaSense, INNOVA 1412i) was used to measure the concentrations of FA.

### Analysis and characterizations

X-ray diffraction (XRD) patterns of the photocatalyst samples were collected using an X-ray diffractometer (PANalytical X’Pert diffractometer) using Cu Kα irradiation. Nitrogen adsorption–desorption isotherms were recorded at 77 K by using a BELSORP-MINI II (BEL-Japan, Inc.). Before the measurement, the sample was degassed at 423 K overnight. The specific surface area was calculated via a multipoint BET analysis of the N_2_ adsorption isotherm. FE-SEM images were taken by JSM-7800 F prime microscope at National Institute for Nanomaterials Technology (NINT, Pohang, Korea). X-ray photoelectron spectroscopy (XPS) was conducted using a Thermo Scientific K-Alpha XPS with Al Kα (*hν* = 1486.6 eV) as the excitation source. Fourier transform infrared (FTIR) spectra were obtained using an attenuated total reflectance-FTIR (ATR-FTIR) spectrometer (Thermo Scientific Nicolet iS50 FT–IR/ATR). Diffuse reflectance UV–visible absorption spectroscopy (DRS) was conducted using a spectrophotometer (Shimadzu UV-2401PC).

The quantitative analysis of iodine species, mainly iodate (IO_3_^−^) and periodate (IO_4_^−^) was carried out using HPLC (Agilent 1100) equipped with an Agilent Zorbax 300SB C-18 column and a diode-array detector. The typical eluent consisted of a binary mixture of 0.1% (v/v) phosphoric acid aqueous solution and acetonitrile (typically 70:30 v/v). The photocatalytic production of OH radicals was confirmed using an electron paramagnetic resonance (EPR) spin trapping method (ELEXYS E580, Bruker Co.): 10 mM DMPO as a spin-trapping agent was added to the aqueous catalyst slurry under irradiation of blue LED (Luna Fiber Optic Korea, CWL 460 nm, 2.0 mW/cm^2^). Real-time monitoring of the dynamic movement of catalyst particles and in situ formed water layer in the PA/WO_3_ system was performed using a Leica DM 5000 B microscope equipped with a Leica DFC420 camera (Leica, Wetzlar, Germany).

In situ DRIFTS was performed using an FT-IR spectrometer (PerkinElmer, USA) equipped with a diffuse-reflectance cell (PIKE) with a ZnSe window. The catalyst sample was placed in the cell and pretreated at 100 °C to eliminate the effects of adsorbed water. AA of 300 ppmv was introduced into the cell by diluting the standard gas (1000 ppmv AA in N_2_) with air. The RH was adjusted by bubbling the air through a stainless-steel bottle containing deionized water. In situ DRIFTS spectra were collected after exposing the samples to the flowing stream for 10 s, 1, 3, and 5 min, respectively.

### Computational details

Density function theory (DFT) calculations were performed by using the CP2K package^66^. PBE functional^67^ with Grimme D3 correction^68^ was used to describe the system. Unrestricted Kohn–Sham DFT has been used as the electronic structure method in the framework of the Gaussian and plane waves method^69^. The Goedecker–Teter–Hutter (GTH) pseudopotentials^70^, DZVPMOLOPT-GTH basis sets^69^ were utilized to describe the molecules. A plane-wave energy cut-off of 500 Ry has been employed.

The charge density difference is defined as Eq. (6):

6 $$\Delta \rho ={\rho }_{{{{{{\rm{mol}}}}}}}{/}_{{{{{{\rm{sur}}}}}}}-{\rho }_{{{{{{\rm{mol}}}}}}}-{\rho }_{{{{{{\rm{sur}}}}}}}$$

where *ρ*_mol_/_sur_, *ρ*_mol,_ and *ρ*_sur_ are the electron density of the molecule adsorbed on the surface, the molecule, and the surface, respectively. The BE is defined as Eq. (7):

7 $${E}_{{{{{{\rm{b}}}}}}}={E}_{{{{{{\rm{mol}}}}}}}{/}_{{{{{{\rm{sur}}}}}}}-{E}_{{{{{{\rm{mol}}}}}}}-{E}_{{{{{{\rm{sur}}}}}}}$$

where *E*_mol_/_sur_, *E*_mol_ and *E*_sur_ are the energy of the molecule adsorbed on the surface, the molecule, and the surface, respectively.

### Calculation of apparent quantum efficiency (AQE)

For the measurement of AQE, two monochromatic LEDs (Luna Fiber Optic Korea, 2.0 mW/cm^2^) which emit light at ca. 365 and 460 nm were used in the PCD of AA. Except for the light source, all the conditions were the same as that in the PCD tests using the mercury lamp. The AQE of AA degradation was indirectly calculated based on the CO_2_ generation rate within 40 min PCD reaction to rule out the effect of AA adsorption on the catalyst surface and reactor surface. The PCD of AA can be divided into the following two half-reactions (Eqs. (8)–(10)).

8 $${{{{{\rm{Oxidation}}}}}}\!\!:\,{{{{{{\rm{CH}}}}}}}_{3}{{{{{\rm{CHO}}}}}}+3{{{{{{\rm{H}}}}}}}_{2}{{{{{\rm{O}}}}}}\to 2{{{{{{\rm{CO}}}}}}}_{2}+10{{{{{{\rm{H}}}}}}}^{+}+10{{{{{{\rm{e}}}}}}}^{-}$$

9 $${{{{{\rm{Reduction}}}}}}\!\!:\,2.5{{{{{{\rm{O}}}}}}}_{2}+10{{{{{{\rm{H}}}}}}}^{+}+10{{{{{{\rm{e}}}}}}}^{-}\to 5{{{{{{\rm{H}}}}}}}_{2}{{{{{\rm{O}}}}}}\qquad\qquad\quad$$

10 $$\,{{{{{\rm{Overall}}}}}}\!\!:\,{{{{{{\rm{CH}}}}}}}_{3}{{{{{\rm{CHO}}}}}}+2.5{{{{{{\rm{O}}}}}}}_{2}\to 2{{{{{{\rm{CO}}}}}}}_{2}+2{{{{{{\rm{H}}}}}}}_{2}{{{{{\rm{O}}}}}}\quad\quad$$

The full degradation of an AA molecule leads to the production of two CO_2_ molecules, which is a 10-electron oxidation. Therefore, the production of one CO_2_ molecule needs a 5-electron oxidation, which should consume 5 photons: the AQE estimated from the CO_2_ production measurement is based on this stoichiometry. The number of incident photons indicates the total number of photons reaching the surface of the catalyst during the reaction time, which were calculated by Eq. (11). The number of reacted photons indicates the number of photons that are utilized in transforming AA molecules into CO_2_, which was calculated by Eq. (12). The AQE was calculated by Eq. (12)/Eq. (11). Such estimation of AQE is based on the assumption that AA molecules are fully mineralized to CO_2_ with the negligible generation of intermediates. As the PCD of AA on PA/WO_3_ accompanied the stoichiometric production of CO_2_ (see Figs. 1, 3, 5), the formation of intermediates (if any) should be negligible in terms of the carbon mass balance.

11 $${{{{{\rm{Number}}}}}}\,{{{{{\rm{of}}}}}}\,{{{{{\rm{incident}}}}}}\,{{{{{\rm{photons}}}}}}=\frac{{E}{\lambda }{At}}{{hc}}$$

12 $${{{{{\rm{Number}}}}}}\,{{{{{\rm{of}}}}}}\,{{{{{\rm{reacted}}}}}}\,{{{{{\rm{photons}}}}}}=5{R}_{{{{{{{\rm{CO}}}}}}}_{2}}{N}_{{\rm {A}}}t$$

13 $${{{{{\rm{AQE}}}}}}=\frac{{{{{{\rm{Number}}}}}}\,{{{{{\rm{of}}}}}}\,{{{{{\rm{reacted}}}}}}\,{{{{{\rm{photons}}}}}}}{{{{{{\rm{Number}}}}}}\,{{{{{\rm{of}}}}}}\,{{{{{\rm{incident}}}}}}\,{{{{{\rm{photons}}}}}}}\times 100 \%$$

where *E* is the intensity of LED (mW/cm^2^); *λ* is the wavelength of LED; *A* is the irradiation area of photocatalysts; *t* is the illumination/reaction time (40 min); *h* is the Planck constant, 6.626 × 10^−34^ Js; *c* is the velocity of light; *R*_CO2_ is the CO_2_ generation rate (mol/min); and *N*_A_ is the Avogadro constant, 6.02 × 10^23^/mol.

## Supplementary information

Supplementary Information

Peer Review File

Description of Additional Supplementary Files

Supplementary Video 1

## Data Availability

Source data are provided with this paper. All data are also available from the corresponding author on request.

## Source data

Source Data
