# Supplementary material for: Self-wetting triphase photocatalysis for effective and selective removal of hydrophilic volatile organic compounds in air
Source: Nat Commun. 2021 Oct 29;12:6259. doi: 10.1038/s41467-021-26541-z (PMC8556241; doi:10.1038/s41467-021-26541-z)
Supplement: Supplementary file 1 — Supplementary Information [file 41467_2021_26541_MOESM1_ESM.pdf]

Supplementary information

Self-wetting triphase photocatalysis for efficient and selective removal of hydrophilic volatile organic compounds in air

Fei He[1], Seunghyun Weon[2], Woojung Jeon[1], Myoung Won Chung[2] & Wonyong Choi[1,*]

[1]Division of Environmental Science and Engineering, Pohang University of Science and Technology (POSTECH), 37673, Pohang, Korea

[2]School of Health and Environmental Science, Korea University, 02841, Seoul, Korea

*Corresponding author. E-mail: wchoi@postech.edu; Fax: +82-54-279-8299

Supplementary Figures and Tables

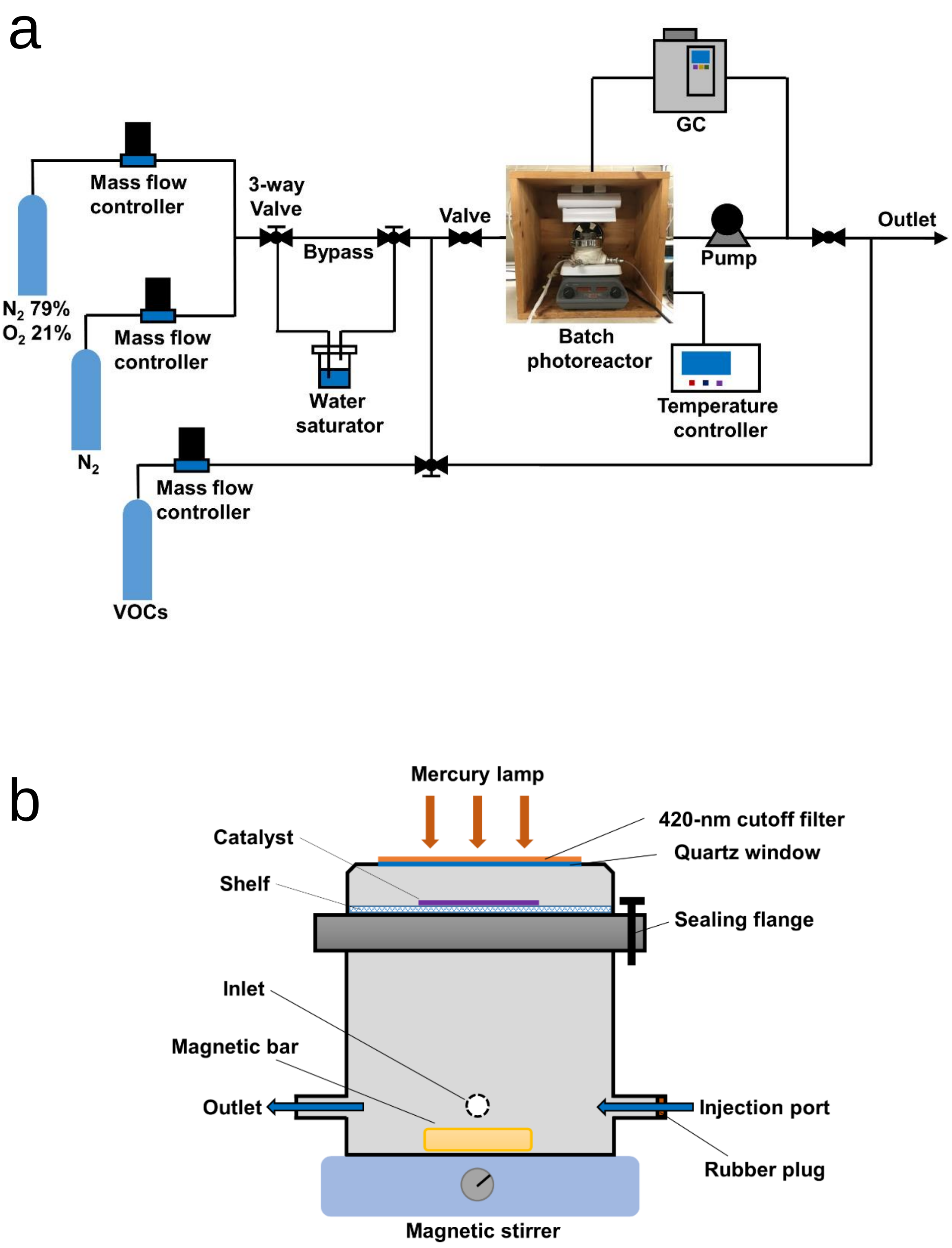

Supplementary Figure 1. Schematics of the reactor system. a The experimental set-up. **b** The batch photoreactor.

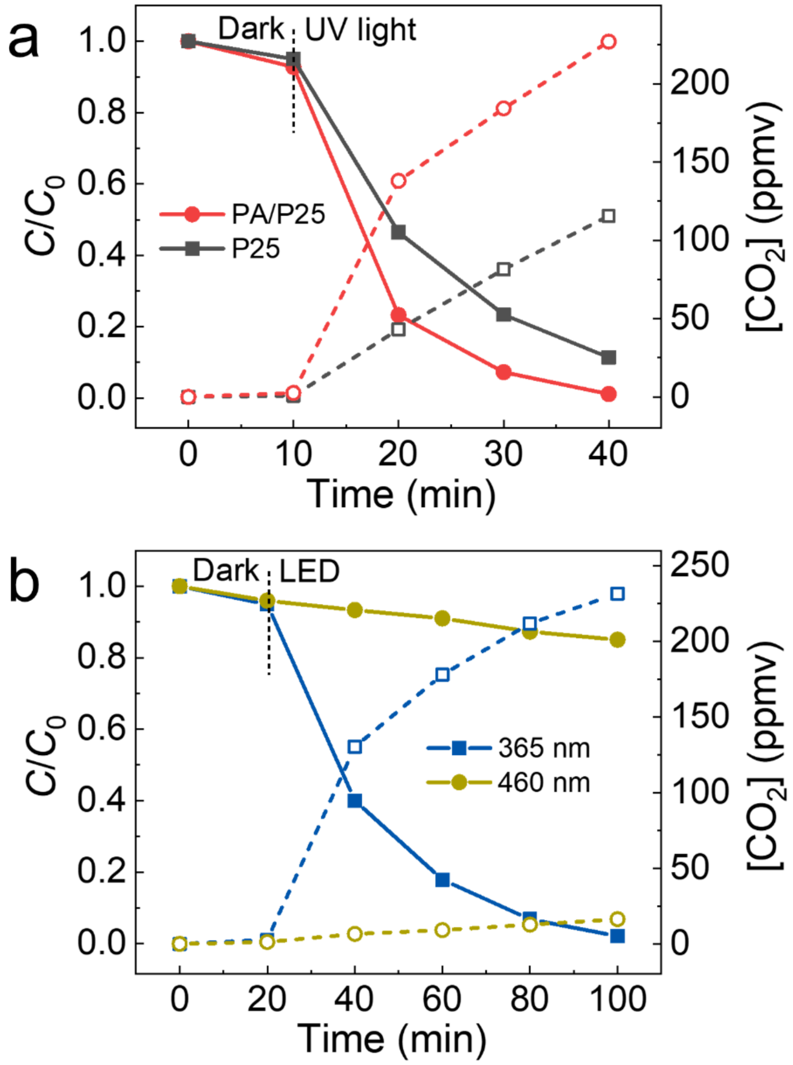

Supplementary Figure 2. PCD of acetaldehyde (AA) in TiO_2/UV system. PCD activities for **a** PA/P25 and P25 under irradiation of UV mercury lamp (13.3 mW/cm^2) and for **b** PA/P25 under irradiation of LED (λ = 460 nm or 365 nm, 2.0 mW/cm^2). The dashed lines with open symbols represent the CO_2 concentration generated from AA degradation. Experimental conditions: $[AA]_0$ = 120 ppmv; sample amount of 50 mg; RH 65%; reaction temperature of 30 ^{o}C.

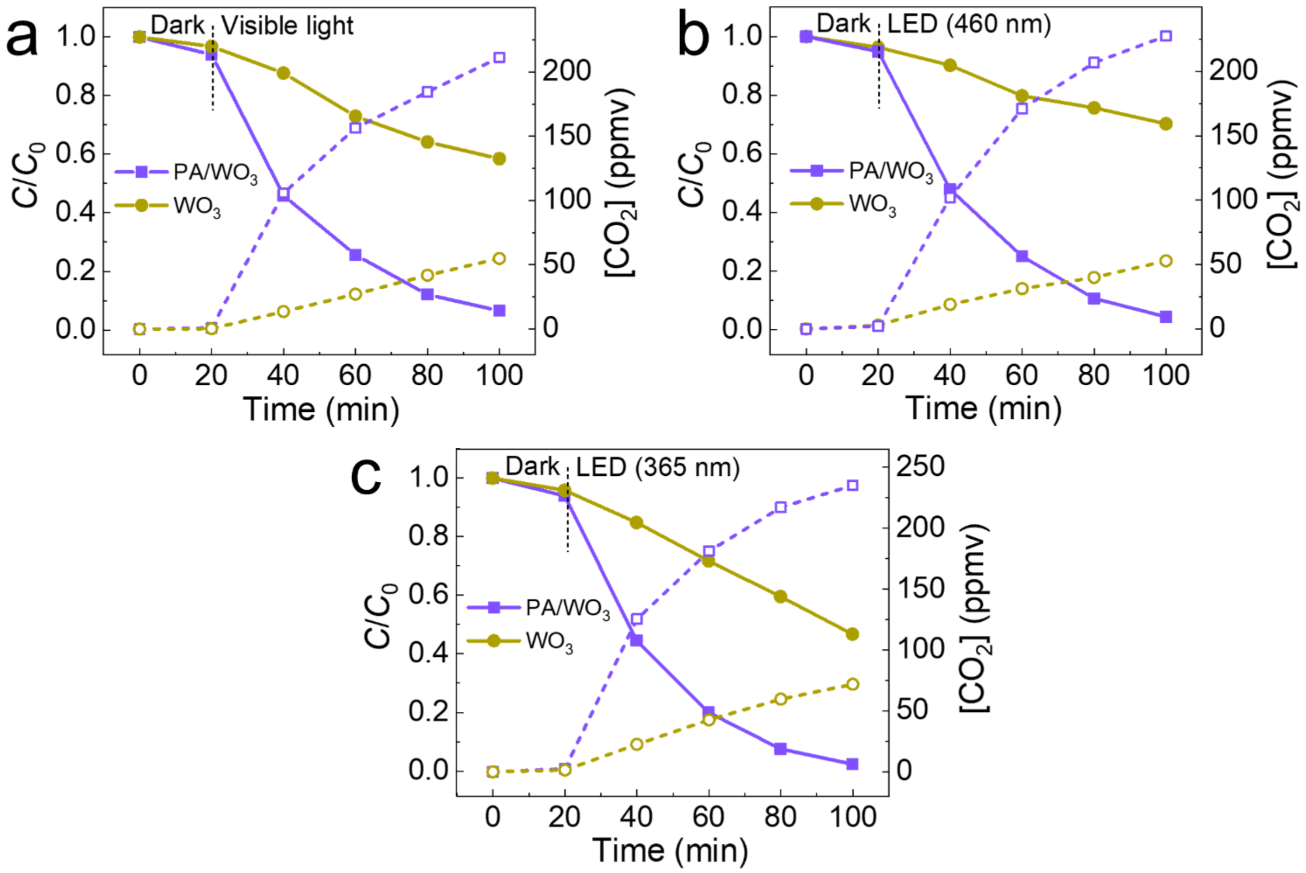

Supplementary Figure 3. PCD activities for PA/WO$_3$ and bare WO$_3$ under various light sources. **a** Halogen lamp with a 420-nm cutoff filter (λ > 420 nm, 7.5 mW/cm^2). **b** Blue LED (λ = 460 nm, 2.0 mW/cm^2). **c** UV LED (λ = 365 nm, 2.0 mW/cm^2). The dashed lines with open symbols represent the CO_2 concentration generated from AA degradation. Experimental conditions: $[AA]_0$ = 120 ppmv; sample amount of 50 mg; RH 65%; reaction temperature of 30 ^{o}C.

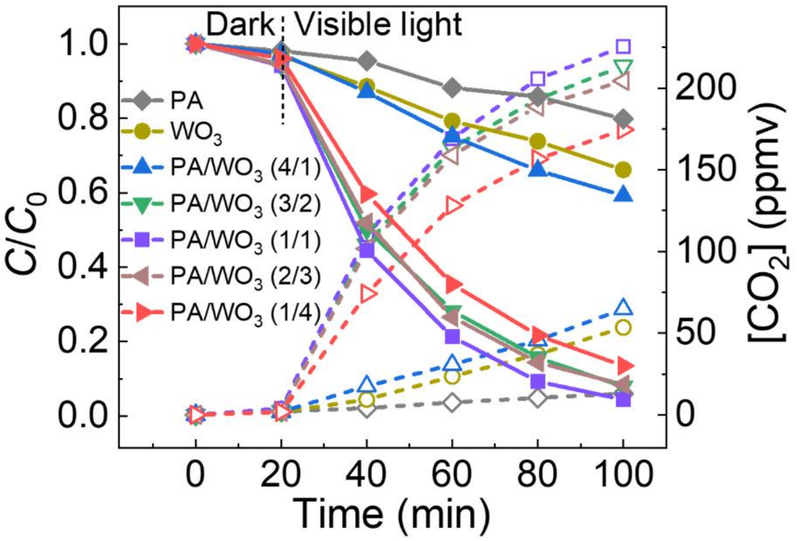

Supplementary Figure 4. PCD activity of PA/WO_3 with different PA:WO_3 mass ratio (*w*/*w*) for acetaldehyde (AA) degradation. The dashed lines with open symbols represent CO_2 generated from AA degradation. Experimental conditions: $[AA]_0$ = 120 ppmv; visible light (λ > 420 nm) intensity of 2.2 mW/cm^2; the sample mass (PA+WO_3) fixed at 50 mg for all samples; RH 65%; reaction temperature of 30 ^{o}C.

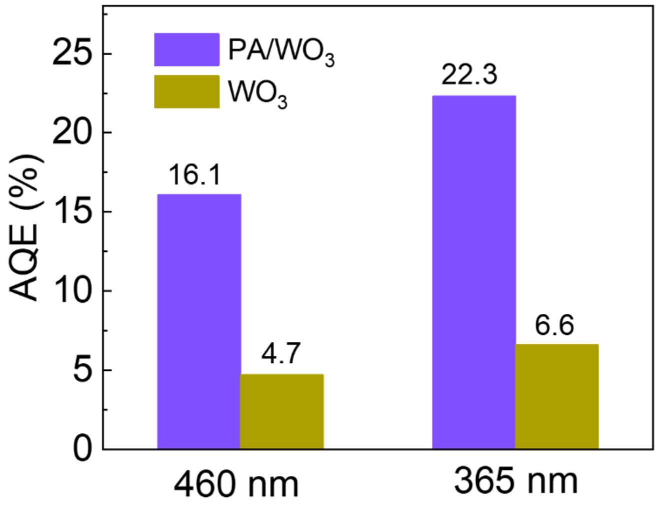

Supplementary Figure 5. The apparent quantum efficiency (AQE) of PA/WO_3 and bare WO_3 in the PCD of acetaldehyde (AA) under blue LED (λ = 460 nm) and UV LED (λ = 365 nm) irradiation. Experimental conditions: $[AA]_0$ = 120 ppmv; light intensity of 2.0 mW/cm^2; sample amount of 50 mg; RH 65%; reaction temperature of 30 ^{o}C.

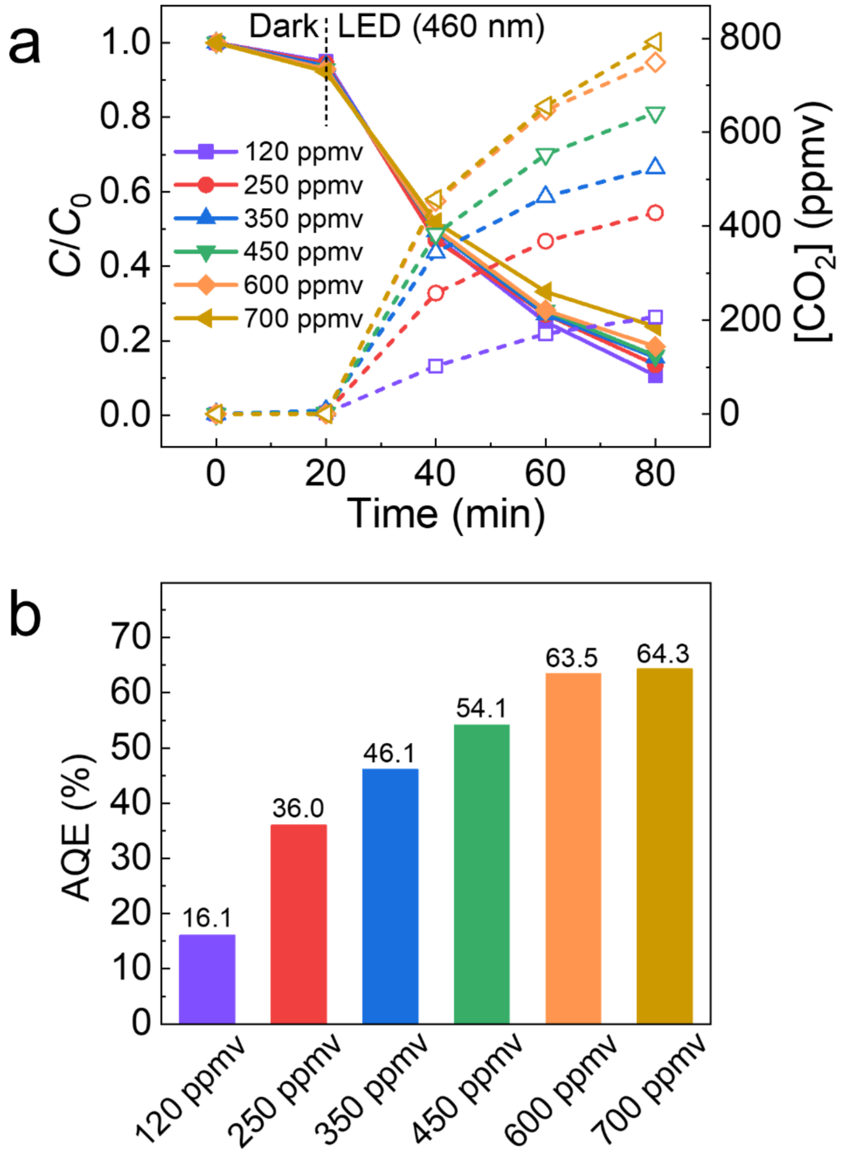

Supplementary Figure 6. The activities of PA/WO_3 in the PCD of acetaldehyde (AA) with different initial concentrations (C_0) under blue LED (λ = 460 nm) irradiation. a The time profiles of the PCD of AA and the accompanying production of CO_2. **b** Apparent quantum efficiency (AQE) of PA/WO_3. The dashed lines with open symbols represent the CO_2 concentration generated from AA degradation. Experimental conditions: light intensity of 2.0 mW/cm^2; sample amount of 50 mg; RH 65%; reaction temperature of 30 ^oC.

To investigate the degradation potential of PA/WO_3 on air purification, AA in different initial concentrations ($[AA]_0$ = 120 ppmv-700 ppmv) was introduced in the PCD reactor. As shown above, most AA could be removed within 80 min PCD even at $[AA]_0$ = 700 ppmv. The AQEs were estimated based on the production of CO_2 (Eqs. 11-13). With increasing $[AA]_0$, AQE continued to increase until it reached a plateau at 600 ppmv. The AQE is as high as 64.3% at 700 ppmv.

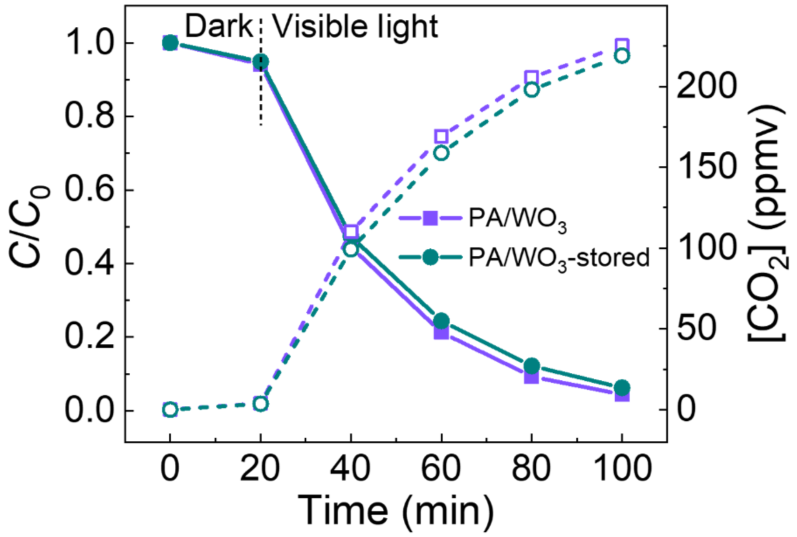

Supplementary Figure 7. PCD activities of fresh PA/WO_3 and PA/WO_3 stored for six months. The dashed lines with the open symbols represent the CO_2 generated from AA degradation. Experimental conditions: $[AA]_0$ = 120 ppmv; visible light (λ > 420 nm) intensity of 2.2 mW/cm^2; sample amount of 50 mg; RH 65%; reaction temperature of 30 ^{o}C.

The PCD activity of PA/WO_3 stored under ambient conditions for six months (denoted as PA/WO_3-stored) was tested after drying at 80 ^{o}C. It was found that the PA/WO_3-stored still has good hygroscopicity and its photocatalytic activity is almost the same with that of fresh PA/WO_3, indicating that the sample remained stable in the long-term storage.

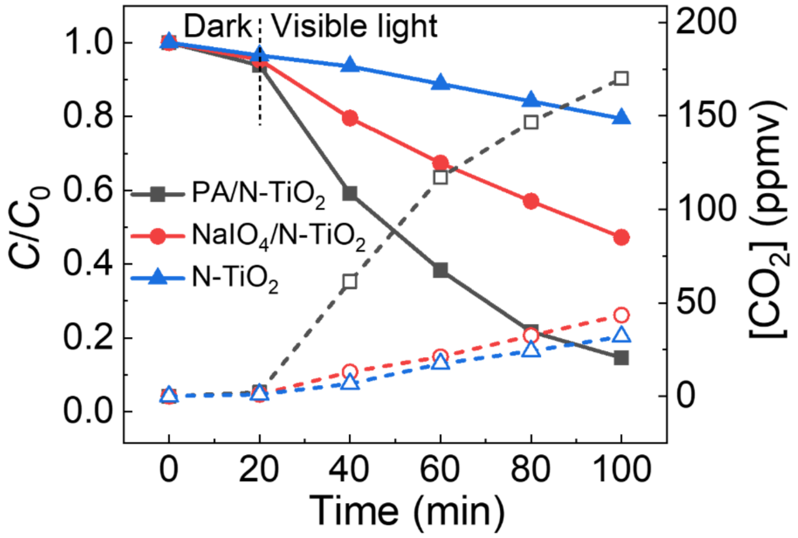

Supplementary Figure 8. PCD activities of PA/N-TiO_2, $NaIO_4$/N-TiO_2 and N-TiO_2. The dashed lines with open symbols represent the CO_2 concentration generated from AA degradation. Experimental conditions: $[AA]_0$ = 120 ppmv; visible light (λ > 420 nm) intensity of 2.2 mW/cm^2; sample amount of 50 mg; RH 65%; reaction temperature of 30 ^oC.

In order to further confirm the promoting effect of PA, another classic visible-light driven photocatalyst (N-TiO_2) was selected to combined with PA or $NaIO_4$ following the same procedure as in the preparation of PA/WO_3 or $NaIO_4$/WO_3. The PCD activity of $NaIO_4$/N-TiO_2 (k_d = 9.51 × 10^{-3} min^{-1}) was much lower than that of PA/N-TiO_2 (k_d = 24.63 × 10^{-3} min^{-1}). PA has a much better promoting effect than $NaIO_4$ regardless of the kind of photocatalysts.

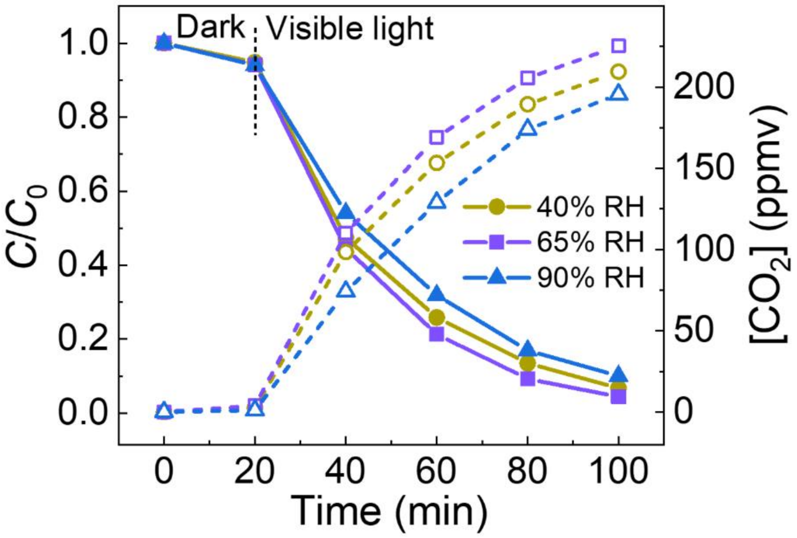

Supplementary Figure 9. PCD activity of PA/WO_3 for acetaldehyde (AA) degradation in humid air with different relative humidity (RH). The dashed lines with open symbols represent CO_2 generated from AA degradation. Experimental conditions: $[AA]_0 = 120$ ppmv; visible light ($\lambda > 420$ nm) intensity of 2.2 mW/cm^2; sample amount of 50 mg; reaction temperature of 30 ^oC.

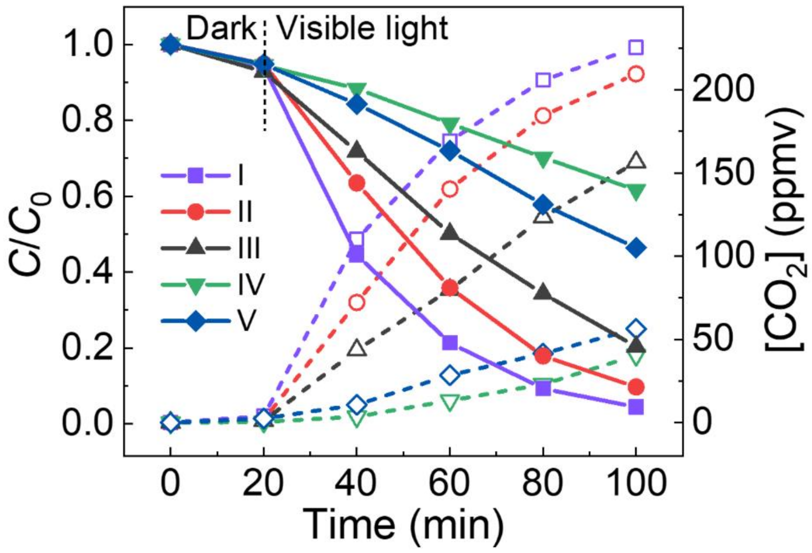

Supplementary Figure 10. PCD activities of (I) PA/WO_3, (II) acidified $NaIO_4$/WO_3 slurry (iodic acid was used to adjust pH to 1.5), (III) $NaIO_4$/WO_3 slurry, (IV) HIO_3/WO_3 slurry and (V) WO_3 slurry. The dashed lines with open symbols represent the CO_2 generated from acetaldehyde (AA) degradation. Experimental conditions: all slurry samples containing 25 mg WO_3 and 13 mg water; $[AA]_0$ = 120 ppmv; visible light (λ > 420 nm) intensity of 2.2 mW/cm^2; RH 65%; reaction temperature of 30 ^{o}C.

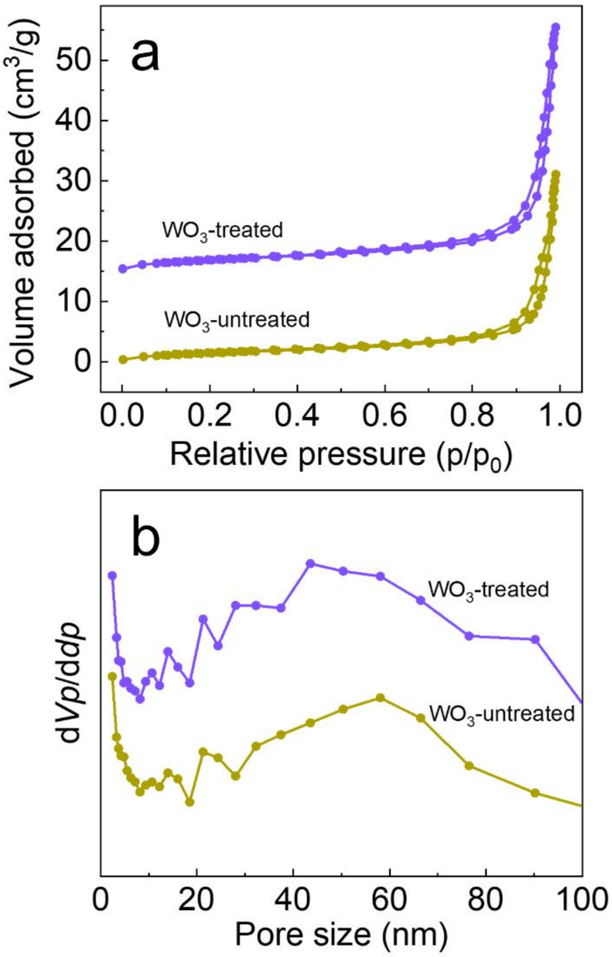

Supplementary Figure 11. N_2 absorption-desorption experiment results of WO_3-treated and WO_3-untreated. a N_2 adsorption–desorption isotherms. **b** Pore-size distributions. The data curves of the WO_3-treated sample were shifted upwards to avoid overlapping for clarity.

PA treatment may affect the intrinsic physicochemical properties of WO_3 due to the acidic environment it provides or the load of surface functional groups. N_2 absorption-desorption experiments, FE-SEM and TEM were employed to investigate the pore structure and morphology changes of WO_3 before and after PA treatment. Before the test, PA/WO_3 was washed thoroughly with water (denoted as WO_3-treated) to eliminate the interference of abundant PA to the results. Then, its N_2 adsorption-desorption isotherms and pore-size distributions were compared with bare WO_3 (denoted as WO_3-untreated). As can be seen in Supplementary Figure 11, both the two samples showed a type II isotherm with an H3 hysteresis loop at relative pressure (P/P_0) range of 0.8–1.0. This indicated that there are slit-shaped pores in the samples. Usually, mesoporous/macroporous could be formed in solids consisting of aggregated or agglomerated particles with nonuniform size and/or shape. This can be confirmed by the irregular pore size distribution of both samples as shown in Supplementary Figure 11b. The S_{BET} and average pore size of WO_3-untreated were 5.65 m^2/g and 33.6 nm respectively, and those of WO_3-treated were 7.27 m^2/g and 34.2 nm respectively. Compared with the untreated WO_3, no significant changes were detected in the nature of isotherm, BET surface area (S_{BET}) and total pore volume for the WO_3 after PA processing.

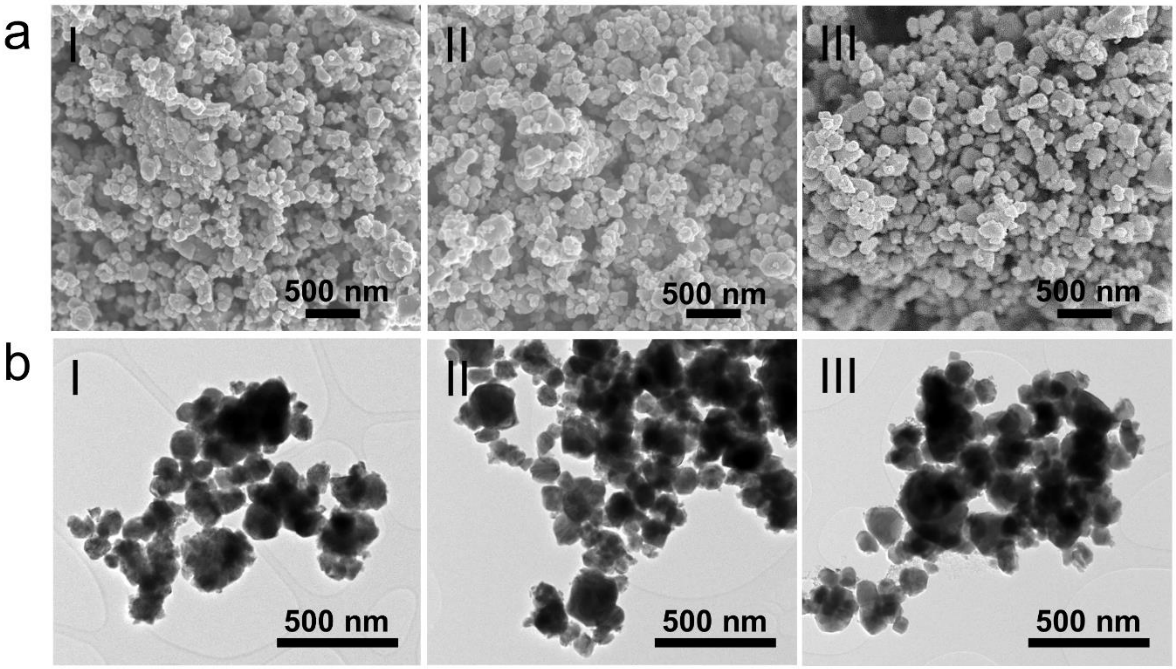

Supplementary Figure 12. The morphology of photocatalysts. a FE-SEM and **b** TEM image of (I) WO_3-untreated, (II) WO_3-treated and (III) WO_3-used.

The morphology of WO_3-treated and WO_3-untreated were observed by FE-SEM and TEM. The morphology of untreated WO_3 was irregular nanoparticles with variable sizes ranging from 20 to 100 nm. No obvious difference was observed in the FE-SEM and TEM image of the treated WO_3, indicating that the PA treatment processing negligibly affected the size and morphology of WO_3. The possible morphology changes of WO_3 after the PCD of AA was also investigated using FE-SEM and TEM. To eliminate the interference of abundant PA to the results, the PA/WO_3 used after PCD of AA was washed thoroughly with water (denoted as WO_3-used). As shown above, no obvious change was found in the morphology of WO_3-used compared with that of fresh WO_3 (namely WO_3-untreated), indicating that the size and morphology of WO_3 were little changed during the PCD reaction of AA.

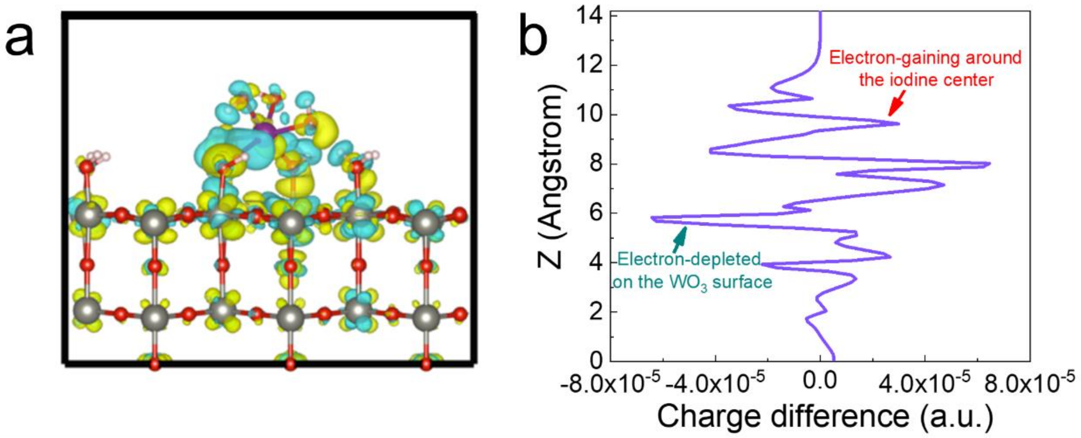

Supplementary Figure 13. Charge density difference in PA/WO_3 system. a The 3-dimensional iso-density of charge density difference. Color code: tungsten (gray), oxygen (red), iodine (purple), hydrogen (white). Isosurface: yellow color indicates the gaining of electron density while cyan color indicates the losing of electron density. **b** The x–y plane averaged charge difference with charge density (ρ) on the x-axis and the distance (Å) in z-direction of the interface unit cell on the y-axis.

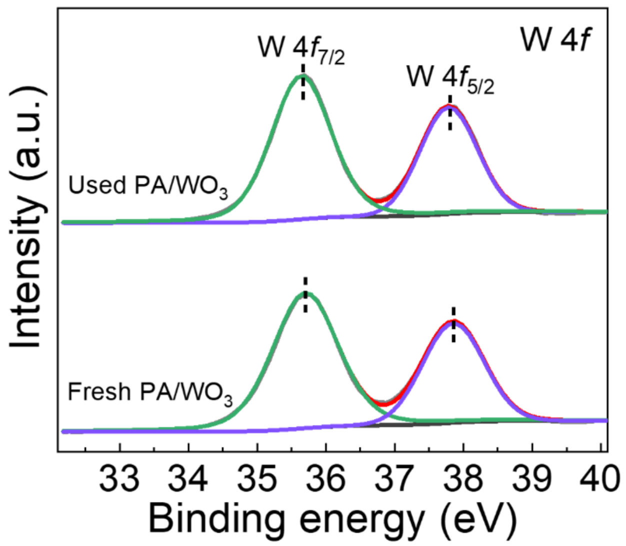

Supplementary Figure 14. W 4*f* XPS spectra of the fresh and used PA/WO_3 samples.

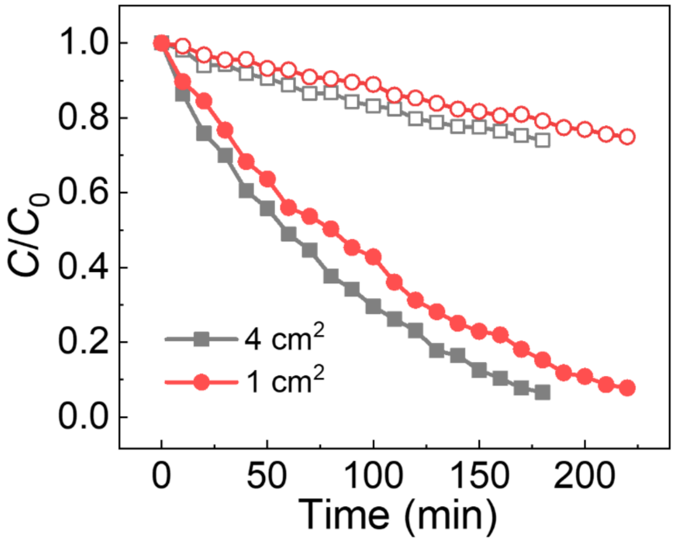

Supplementary Figure 15. The PCD of formaldehyde (FA) (filled symbols) at 500 ppbv over 1 mg PA/WO_3 coated on the area of 4 cm^2 (2 cm × 2 cm) *versus* 1 cm^2 (1 cm × 1 cm). The dark control tests (open symbols) are also compared. Experimental conditions: $[FA]_0$ = 500 ppbv; reactor volume of 1.5 L; blue LED (λ = 460 nm) intensity of 2.0 mW/cm^2; RH 65%; reaction temperature of 30 ^oC.

Supplementary Table 1. Photocatalytic degradation of AA over various photocatalyst samples under visible light (λ > 420 nm)

Sample	k_d[a] ($\times 10^{-3}$ min^{-1})	Removal[b] (%)	Mineralization efficiency[c] (%)
PA	2.77 (±) 0.10	20.2 (±) 4.4	5.5 (±) 0.7
$BiVO_4$	3.41 (±) 0.34	23.4 (±) 1.5	4.0 (±) 1.0
N-TiO_2	2.91 (±) 0.16	20.6 (±) 1.7	13.3 (±) 2.3
WO_3	5.26 (±) 0.57	33.8 (±) 3.4	22.3 (±) 3.5
PA/$BiVO_4$	8.19 (±) 1.10	46.7 (±) 4.8	24.5 (±) 2.6
PA/N-TiO_2	24.63 (±) 2.44	85.4 (±) 3.3	70.9 (±) 4.1
**PA/WO_3**	**39.30 (±) 1.84**	**95.6 (±) 0.03**	**93.9 (±) 2.6**
NaI/WO_3	2.16 (±) 0.91	13.7 (±) 3.7	3.6 (±) 0.6
HIO_3/WO_3	3.47 (±) 0.33	23.3 (±) 3.4	10.2 (±) 2.7
$NaIO_4$/WO_3	9.65 (±) 1.16	52.1 (±) 3.3	38.7 (±) 2.3
$NaIO_3$/WO_3	5.29 (±) 0.08	34.8 (±) 3.0	13.7 (±) 2.9
Pt(1%)/WO_3	14.35 (±) 1.06	69.4 (±) 2.6	23.4 (±) 3.6

[a] k_d was calculated by fitting the results of Figure 1 to the pseudo-first-order equation
[b] Removal(%) = ($\Delta[CH_3CHO]/[CH_3CHO]_0$)×100 (after 80 min reaction)
[c] Conversion to CO_2(%) = ($\Delta[CO_2]/2[CH_3CHO]_0$)×100 (after 80 min of reaction)

Supplementary Table 2. Photocatalytic degradation of AA over PA/WO_3 and bare WO_3 under the irradiation of different lamps

Lamp	k_d[a] ($\times 10^{-3}$ min^{-1})		Removal[b] (%)		Mineralization efficiency[c] (%)	
	PA/WO_3	WO_3	PA/WO_3	WO_3	PA/WO_3	WO_3
Mercury lamp (λ > 420 nm) (Intensity: 2.2 mW/cm^2)	39.30	5.26	95.6	33.8	93.9	22.3
Halogen lamp (λ > 420 nm) (Intensity: 7.5 mW/cm^2)	34.56	7.07	93.4	41.5	88.0	22.9
LED (λ = 460 nm) (Intensity: 2.0 mW/cm^2)	37.82	4.67	95.6	29.7	94.8	22.1
LED (λ = 365 nm) (Intensity: 2.0 mW/cm^2)	44.70	9.04	97.7	53.3	98.0	30.0

[a] k_d was calculated by fitting the results of Supplementary Figure 3 to the pseudo-first-order equation
[b] Removal(%) = ($\Delta[CH_3CHO]/[CH_3CHO]_0$)×100 (after 80 min reaction)
[c] Conversion to CO_2(%) = ($\Delta[CO_2]/2[CH_3CHO]_0$)×100 (after 80 min of reaction)

Supplementary Table 3. AQE of representative photocatalysts for AA degradation under visible light

Catalyst type	Catalyst amount /Reactor	Light source	AA concentration	AQE	Ref.
$TiO_{2-x}N_x$	–/–	Fuorescent light: λ = 436 nm, I = 0.9 mW/cm^2	485 ppmv	0.42%	[1]
CuO/WO_3	150 mg/4.4 ml (b)#	29 µW xenon lamp with monochromator: λ = 400 nm	Injection: 40 µL gaseous AA	3.2%	[2]
$CaFe_2O_4/WO_3$	0.1 g/0.5 L (b)	150 W Xe lamp with UV-cutoff filter	100 ppmv	1.48%	[3]
Melam/WO_3	100 mg/125 mL (b)	LED: λ = 435 nm, I = 3.0 mW/cm^2	500 ppmv	~2.2%	[4]
N-V_2O_5–ZnO	0.05 g/250 mL (b)	150 W Xe lamp: λ = 470 nm	790 ppmv	~0.6%	[5]
Fe_2O_3/S-TiO_2 nanotubes	100 mg/125 mL (b)	500 W Xe lamp: λ = 420 nm, I = 12.7 mW/cm^2	500 ppmv	2.2%	[6]
0.5 wt % Pt-loaded WO_3 NTs	100 mg/500 mL (b)	300 W Xe lamp: λ > 400 nm, I = 20 mW/cm^2	500 ppmv	1.03%	[7]
0.5 wt % Pt-loaded commercial WO_3 particles	100 mg/500mL (b)	300 W Xe lamp: λ > 400 nm, I = 20 mW/cm^2	500 ppmv	0.24%	[7]
$(Ag_{0.75}Sr_{0.25})(Nb_{0.75}\text{-}Ti_{0.25})O_3$	0.4 g/500mL (b)	Xe arc lamp: λ = 440 nm, I = 30 mW/cm^2	250 ppmv	1.48%	[8]
Bi_2WO_6 particles	50 mg/330 mL (b)	300 W xenon lamp: λ = 400 nm	2000 ppmv	8%	[9]
$ZnBi_{12}O_{20}$	0.8 g/– (b)	300 W Xe arc lamp: λ = 400 nm	837 ppmv	0.25%	[10]
F-doped TiO_2	0.05 g/–(b)	150 W Xe lamp: λ > 420 nm	930 ppmv	0.55%	[11]
**PA/WO_3**	**50 mg/300 mL (b)**	**LED: λ = 460 nm, I = 2.0 mW/cm^2**	**120 ppmv**	**16.1 %**	**This work**
**PA/WO_3**	**50 mg/300 mL (b)**	**LED: λ = 460 nm, I = 2.0 mW/cm^2**	**700 ppmv**	**64.3%**	**This work**

(b) stands for batch reactor.

Supplementary Table 4. WO_3-based photocatalysts for typical VOCs degradation under visible light

Catalyst type	Catalyst amount /Reactor	VOC type/concentration	Light source	AQE	Ref.
WO_3/TiO_2 nanotubes	2 cm^2 film/15 mL (b)#	Isopropanol/165 ppmv	LED: λ = 400 nm, I = 112 mW/cm^2	0.35%	[12]
CuO/WO_3	150 mg/4.4 ml (b)	Acetic acid/ Evaporation: 2 µL liquid acetic acid	29 µW xenon lamp with monochromator: λ = 400 nm	6.3%	[2]
CuO/WO_3	150 mg/4.4 ml (b)	Formaldehyde/ Evaporation: 2 µL 16 wt % formaldehyde solution	29 µW xenon lamp with monochromator: λ = 400 nm	2.3%	[2]
WC/WO_3	–/– (b)	Isopropanol/300 ppmv	Xe lamp with UV-cutoff filter: λ = 400 -530 nm I = 0.67 mW/cm^2	3.2%	[13]
Pt/WO_3	Suspension catalyst (5 g/L) /330 mL (b)	Acetic acid/–	300 W xenon lamp with monochromator: λ = 400 nm	~10%	[14]
**PA/WO_3**	**50 mg/300 mL (b)**	**120 ppmv**	**LED: λ = 460 nm, I = 2.0 mW/cm^2**	**16.1 %**	**This work**
**PA/WO_3**	**50 mg/300 mL (b)**	**700 ppmv**	**LED: λ = 460 nm, I = 2.0 mW/cm^2**	**64.3%**	**This work**

(b) stands for batch reactor.

In general, it is difficult to make a direct comparison of the performance of photocatalysts, because the efficiency depends on the experimental conditions such as light intensity, reactor type, and substrate concentration. AQE is usually a good indicator to compare the activity of photocatalysts because it can normalize different light intensity conditions. Nevertheless, AQE is still affected by the type of pollutants and light conditions. For example, Arai et al. found that the AQEs in the PCD of acetic acid, acetaldehyde, formaldehyde on CuO/WO_3 were significantly different (J. Phys. Chem. C 2009, 113, 6602). Therefore, the AQEs obtained in this work are compared with some representative visible-light driven PCD systems for AA degradation in the literature (Supplementary Table 3). The AQE of PA/WO_3 (64.3%) is the highest value ever reported in the PCD of AA under visible light irradiation. Besides, the PCD activities of some typical WO_3-based catalysts for VOCs other than AA are also listed in Supplementary Table 4. PA/WO_3 is also remarkable among these visible-light driven WO_3

PCD systems. This fully demonstrates the advantages of the reported PA/WO_3 as a triphase gas-liquid-solid photocatalytic system over the traditional two-phase gas-solid photocatalytic system in treating AA. The superior photocatalytic activity can be attributed to the enrichment of hydrophilic VOCs in the in-situ surface water layer, and the efficient electron scavenging by PA. These findings were not unique to WO_3, and other PA-coated semiconductors (e.g., N-TiO_2, $BiVO_4$) also showed high performance. This proposes a low-cost and facile way to efficiently eliminate hydrophilic VOCs under visible light.

Supplementary References:

1 Asahi, R., Morikawa, T., Ohwaki, T., Aoki, K. & Taga, Y. Visible-light photocatalysis in nitrogen-doped titanium oxides. *Science* **293**, 269-271 (2001).

2 Arai, T., Horiguchi, M., Yanagida, M., Gunji, T., Sugihara, H. & Sayama, K. Reaction mechanism and activity of WO_3-catalyzed photodegradation of organic substances promoted by a CuO cocatalyst. *J. Phys. Chem. C* **113**, 6602-6609 (2009).

3 Miyauchi, M., Nukui, Y., Atarashi, D. & Sakai, E. Selective growth of n-type nanoparticles on p-type semiconductors for Z-scheme photocatalysis. *ACS Appl. Mater. Interfaces* **5**, 9770-9776 (2013).

4 Jin, Z. Y., Zhang, Q. T., Hu, L., Chen, J. Q., Cheng, X., Zeng, Y. J., Ruan, S. C. & Ohno, T. Constructing hydrogen bond based melam/WO_3 heterojunction with enhanced visible-light photocatalytic activity. *Appl. Catal., B.* **205**, 569-575 (2017).

5 Li, D. & Haneda, H. Enhancement of photocatalytic activity of sprayed nitrogen-containing ZnO powders by coupling with metal oxides during the acetaldehyde decomposition. *Chemosphere* **54**, 1099-1110 (2004).

6 Nishijima, K., Fujisawa, Y., Murakami, N., Tsubota, T. & Ohno, T. Development of an S-doped titania nanotube (TNT) site-selectively loaded with iron(III) oxide and its photocatalytic activities. *Appl. Catal., B.* **84**, 584-590 (2008).

7 Zhao, Z. G. & Miyauchi, M. Nanoporous-walled tungsten oxide nanotubes as highly active visible-light-driven photocatalysts. *Angew. Chem. Int. Ed.* **47**, 7051-7055 (2008).

8 Wang, D. F., Kako, T. & Ye, J. H. Efficient photocatalytic decomposition of acetaldehyde over a solid-solution perovskite $(Ag_{0.75}Sr_{0.25})(Nb_{0.75}Ti_{0.25})O_3$ under visible-light irradiation. *J. Am. Chem. Soc.* **130**, 2724-2725 (2008).

9 Amano, F., Yamakata, A., Nogami, K., Osawa, M. & Ohtani, B. Visible light responsive pristine metal oxide photocatalyst: Enhancement of activity by crystallization under hydrothermal treatment. *J. Am. Chem. Soc.* **130**, 17650-17651 (2008).

10 Tang, J. W. & Ye, J. H. Photocatalytic and photophysical properties of visible-light-driven photocatalyst $ZnBi_{12}O_{20}$. *Chem. Phys. Lett.* **410**, 104-107 (2005).

11 Li, D., Haneda, H., Labhsetwar, N. K., Hishita, S. & Ohashi, N. Visible-light-driven photocatalysis on fluorine-doped TiO_2 powders by the creation of surface oxygen vacancies. *Chem. Phys. Lett.* **401**, 579-584 (2005).

12 Wang, X. G., Sun, M. H., Murugananthan, M., Zhang, Y. R. & Zhang, L. Z. Electrochemically self-doped WO_3/TiO_2 nanotubes for photocatalytic degradation of volatile organic compounds. *Appl. Catal., B.* **260**, 118205 (2020).

13 Kim, Y., Irie1, H. & Hashimoto, K. A visible light-sensitive tungsten carbide/tungsten trioxde composite photocatalyst, *Appl. Phys. Lett.* **92**, 182107 (2008).

14 Abe, R., Takami, H., Murakami, N. & Ohtani, B. Pristine simple oxides as visible light driven photocatalysts: highly efficient decomposition of organic compounds over platinum-loaded tungsten oxide. *J. Am. Chem. Soc.* **130**, 7780-7781 (2008).
