# Supplementary material for: Self-wetting triphase photocatalysis for effective and selective removal of hydrophilic volatile organic compounds in air
Source: Nat Commun. 2021 Oct 29;12:6259. doi: 10.1038/s41467-021-26541-z (PMC8556241; doi:10.1038/s41467-021-26541-z)
Supplement: Supplementary file 3 — Description of Additional Supplementary Files [file 41467_2021_26541_MOESM3_ESM.pdf]

Description of Additional Supplementary Files

File Name: Supplementary Movie 1
Description: Real-time microscopic monitoring of the in-situ water layer formation and dynamic movement of catalyst particles in the PA/WO3 system.
